# Android malware detection method based on highly distinguishable static features and DenseNet

**DOI:** 10.1371/journal.pone.0276332

**Published:** 2022-11-23

**Authors:** Jiyun Yang, Zhibo Zhang, Heng Zhang, JiaWen Fan

**Affiliations:** The College of Computer Science, Chongqing University, Chongqing, China; Abdul Wali Khan University Mardan, PAKISTAN

## Abstract

The rapid growth of malware has become a serious problem that threatens the security of the mobile ecosystem and needs to be studied and resolved. Android is the main target of attackers due to its open source and popularity. To solve this serious problem, an accurate and efficient malware detection method is needed. Most existing methods use a single type of feature, which can be easily bypassed, resulting in low detection accuracy. In addition, although multiple types of features are used in some methods to solve the drawbacks of detection methods using a single type of feature, there are still some problems. Firstly, due to multiple types of features, the number of features in the initial feature set is extremely large, and some methods directly use them for training, resulting in excessive overhead. Furthermore, some methods utilize feature selection to reduce the dimensionality of features, but they do not select highly distinguishable features, resulting in poor detection performance. In this article, an effective and accurate method for identifying Android malware, which is based on an analysis of the use of seven types of static features in Android is proposed to cope with the rapid increase in the amount of Android malware and overcome the drawbacks of detection methods using a single type of feature. Instead of utilizing all extracted features, we design three levels of feature selection methods to obtain highly distinguishable features that can be effective in identifying malware. Then a fully densely connected convolutional network based on DenseNet is adopted to leverage features more efficiently and effectively for malware detection. Compared with the number of features in the original feature set, the number of features in the feature set obtained by the three levels of feature selection methods is reduced by about 97%, but the accuracy is only reduced by 0.45%, and the accuracy is more than 99% in a variety of machine learning methods. Moreover, we compare our detection method with different machine learning models, and the experimental results show that our method outperforms general machine learning models. We also compare the performance of our detection method with two state-of-the-art neural networks. The experimental results show that our detection model can greatly reduce the training cost and still achieve good detection performance, reaching an accuracy of 99.72%. In addition, we compare our detection method with other similar detection methods that also use multiple types of features. The results show that our detection method is superior to the comparison methods.

## Introduction

With the popularity of smartphones and the continuous improvement in their functions, mobile phones have changed from the initial simple communication tool to an inseparable necessity in people’s daily lives. The Android operating system, which was developed and improved by Google, has attracted tremendous attention since its release and has become the most widely used mobile operating system. According to Statcounter [1], in the second quarter of 2021, the sales of Android mobile devices were very successful, accounting for 72.61% of the market. Specifically, the number of users using Android mobile phones increased from 1.4 billion in 2015 to over 3 billion in 2021, which is an increase of more than 2 times [2]. According to a report [3], there were 3 million apps available for download in Google Play in the first quarter of 2021, and the total number of downloads has exceeded 65 billion thus far. Unfortunately, due to the open source nature of the Android platform, attackers can easily inject malicious code into benign applications to damage the system and steal information. Furthermore, since the censorship of the app store is incomplete, attackers can easily upload their malware to the app store. Wang et al. [4] conducted a large-scale analysis of a total of 6 million apps in 16 Chinese Android app markets and Google Play, and the analysis results showed that about 12.30% of apps in the Chinese Android market were reported as malicious by at least 10 anti-virus engines. Even in Google Play, this figure is still 2.09%. Hence, the Android platform has become the main target of malware developers due to its popularity, open source nature, and imperfect app market censorship. In accordance with the “2018 Android Application Security White Paper” [5], over 3.2 million new Android malware were discovered in 2018, which is an average of more than 458 per hour. In a comparison study by Kaspersky Lab [6] between Q4 of 2020 and Q1 of 2021, only the amount of Adware malware decreases as the amounts of all other malware types increase. It is expected that a large amount of Android malware will continue to be developed and spread to perform various malicious behaviors, including tariff consumption, privacy stealing, and remote control. Moreover, some notorious malicious applications have more than 50 variants, which makes it extremely challenging to detect all of them. Furthermore, unlike other smart mobile device platforms, such as iOS, the Android platform allows users to install applications from unknown sources, such as third-party application markets and websites. Therefore, malware developers can lure or mislead Android users to download malicious or suspicious applications from their servers.

To combat malware, several companies, such as McAfee and Symantec, offer signature-based antivirus products [7] to detect malware. Signature-based approaches extract the unique signature of an application and compare it with existing malicious signatures in the signature database to determine whether the application is malicious or not. Subsequently, heuristic-based detection methods that rely on expert-defined rules to identify malware emerged. Enck et al. [8] proposed Kirin, which can detect malicious applications based on nine permission rules they defined. However, such detection methods have errors caused by people’s subjective biases. In addition, both methods can not effectively detect emerging malware.

To solve the previous problems, intelligent malware detection methods based on machine learning (ML) have received extensive attention. Furthermore, the generalization ability of machine learning algorithms can detect unknown malware well. One of the main types of malware detection methods based on machine learning is to utilize static analysis, which can determine whether an application is malicious without executing it, thus incurring a low overhead for execution. DroidAPIMiner [9] extracted Android malware features at the API level by focusing on critical API calls and performed classification using four commonly used machine learning algorithms. APK Auditor [10] was a permission-based Android malware detection system. It calculated a malware score based on the requested permissions and then calculated the malware threshold limit dynamically using logistic regression (LR). Feizollah et al. [11] evaluated the effectiveness of Android Intents (explicit and implicit), as a feature for identifying malicious apps. They reported that intents are a more valuable feature than permissions in terms of detecting Android malware. However, detection methods that use a single type of feature are easily subverted by some bypass techniques. Nezhadkamali et al. [12] indicated that detection methods using permissions alone are usually counteracted by some means, such as overclaimed permissions, permission escalation attacks, and zero permission attacks. Similarly, Liu et al. [13] pointed out that the use of permissions alone will lead to a high rate of false positives. Therefore, they proposed an approach for detecting malware by combining permissions and API features with resource features and the experiments showed that the accuracy of this method is better than the methods using permissions or API features alone. Shatnawi et al. [14] proposed a detection approach based on permissions and API calls. They used recursive feature elimination to select features. Moreover, Kim et al. [15] reported that different types of features represent different properties of applications. Thus, they built a framework to detect malware using five types of features to reflect various characteristics of applications in various aspects.

Another type of malware detection method is to utilize dynamic analysis, which executes applications in a virtual machine or emulator so that researchers can monitor the running behaviors of applications in real time. Dynamic analysis approaches are mainly divided into two kinds: one is based on taint tracking, and the other is based on system call tracking. TaintDroid [16] is an efficient framework for monitoring Android applications by simultaneously tracing multiple sensitive data sources using a system-wide dynamic tracking technique. DroidTrace [17] is a dynamic analysis tool that utilizes ptrace to observe specific system calls in a target process. It can be seen that dynamic analysis introduces heavy additional overhead due to monitoring the data and behaviors generated by apps at runtime.

Deep learning [18], as a new field of machine learning research, has gained increasing attention in the field of artificial intelligence (AI) and has achieved great success in speech and image recognition. Convolutional Neural Networks (CNNs) are widely used neural network structures, which are trained on massive and well-elucidated datasets. Two problems arise when applying CNNs to Android app classification. One is that these models have a large number of parameters, which causes overfitting easily when the dataset is not large enough. The other is that the networks are not deep enough; thus, more abstract semantic information cannot be extracted.

To achieve rapid detection, static analysis is used in this paper. Most of the previous static detection methods use a single type of feature, and such detection methods are easily bypassed and have a high false positive rate. Even some static approaches that utilize multiple types of features, do not properly process them, resulting in problems such as slow training speed, high model computational overhead, and poor detection performance. In [19–23], various types of features are extracted and directly used in machine learning model or deep learning model for training, resulting in slow training speed and high training overhead. In [24–26], although feature selection is utilized to reduce the dimension of features, the selected features are not able to effectively distinguish between benign and malware, resulting in poor detection performance.

In this paper, we propose a static approach that extracts significant features from apps and uses highly distinguishable information to effectively detect malware using deep learning. The design purpose of our method is to detect malware efficiently and accurately. To overcome the drawbacks of detection methods using a single type of feature and characterize the properties of apps as much as possible, we extract seven key types of static features from apps and then integrate these features into four categories. To obtain highly distinguishable features and reasonably remove the features that are useless for distinguishing between benign and malware, we design three levels of feature selection methods to obtain the optimal feature subset. Specifically, including feature selection based on the mean of weight, correlation analysis based on the Pearson correlation coefficient, and recursive feature selection with cross-validation. Compared with the number of features in the original feature set, the number of features in the feature set obtained by the three-level feature selection method is reduced by about 97%, but the accuracy rate is only reduced by 0.45%, and the accuracy of various machine learning algorithms is above 99%.

Finally, to pursue higher accuracy and model generalization abilities, the selected features are fed into a fully densely connected convolutional network designed based on DenseNet [27] to solve the problems of parameter number increase and gradient disappearance caused by the deeper network.

In summary, our paper makes the following contributions.

- To solve the shortcomings of detection methods using a single type of feature, through relevant research and in-depth exploration of the behavior characteristics of applications, we finally extract seven categories of features to reflect all aspects of the application as comprehensively as possible.
- In this paper, three levels of feature selection methods are designed to obtain features with high discrimination that can effectively solve the problem of an excessive number of features in the feature set caused by multiple types of features while fully guaranteeing the model detection performance.
- In the first level of the feature selection method, considering that different types of features have different characteristics. Therefore, two weight calculation approaches called frequency similarity weight and TF-IDF difference are designed to measure the importance of features.
- In the correlation analysis, we not only analyzed the correlation between features in the same category but also carried out a correlation analysis between features in different categories.
- We construct a fully convolutional network based on DenseNet to solve the problems of a large number of parameters, overfitting, and gradient disappearance caused by the deeper network.
- We design various experiments to verify the effectiveness of our method.

The organization of the rest of the paper is as follows. Section II presents an overview of the related work that has been done in this field. Section III introduces feature processing, including feature extraction, feature classification, and feature selection. Section IV explains the proposed approach in this research. The experimental results are demonstrated in Section V and the conclusion follows in VI.

## Related work

As mentioned earlier, the current research on Android malware detection can be mainly divided into static analysis and dynamic analysis. Detecting Android malware with static analysis, where the application is disassembled to be examined for the presence of any malicious code is a popular approach. Several solutions have been developed using static analysis, which utilizes features such as permissions, API calls, Dalvik byte code, and intents. Yuan et al. [28] utilized TF-IDF to calculate the permission value of each permission and the sensitive value of each application, and then use a machine learning algorithm to detect malware. Alzubi et al. [29] utilized the Harris Hawks Optimization (HHO) algorithm to optimize the hyperparameters of support vector machines to identify malware. Arif JM et al. [30] proposed a permission-based machine learning detection method that utilizes five machine learning classifiers with particle swarm optimization to detect malware. SigPid [31] is a static malware detection system used to manage rapid growth in Android malware; it performs multiple data pruning techniques on the permission information to identify significant permissions that can be effective in distinguishing whether an app is malicious. MaMaDroid [32] abstracted API calls into their package and family and builds Markov chains to detect malware. S3Feature [33] extracted sensitive function call graphs by extending function call graphs, and then extracted sensitive subgraphs and neighbor subgraphs from sensitive function call graphs for malware detection. Lei et al. [34] introduced the notion of a function cluster to automatically transform API calls to a feature vector based on their semantics, making the detection system more resilient to the evolvement of malware. Alazab et al. [35] examined the frequency distribution of permissions and API calls and demonstrate that permissions and API calls play a significant factor in classifying malware variants. MLDroid [36] was an effective and efficient web-based solution that was able to detect malicious apps on the basis of permissions and API calls. Similarly, Sheen et al. [37] proposed an Android malware detection system by considering API calls and permissions as features. They processed these features by using a relief algorithm to train three different classifiers. Yang et al. [38] calculated the permission complement through API calls and requested permissions, and then combined it with API calls to detect malware. Kim et al. [39] developed Scandal, which analyzes the Dalvik byte code of apps and detects privacy leakage in applications. Drebin [40] was a lightweight detection method based on several types of features that enables the identification of malicious apps directly on mobile phones. To show Android behaviors from each app’s AndroidManifest.xml file, parameters such as permissions, actions of components, messages passed with intent, and API calls were extracted by DroidMat [41]. It applies K-means clustering to increase the malware detection capability and classifies the applications as benign or malicious by using the KNN algorithm [42]. Firdaus et al. [24] utilized five machine learning algorithms with genetic search to select the best features, and functional trees give the highest accuracy of 95%. Sandeep et al. [25] chose random forest to perform feature selection on the extracted multiple types of features and used a fully connected deep learning model for malware detection. Bhagwat et al. [43] used Genetic Algorithm (GA), Gravitational Search Algorithm (GSA) and Correlation Genetic Gravitational Search Algorithm (CGGSA) to obtain the optimal feature subset, and then applied Adaptive boosting and Extreme Gradient Boosting Classifiers to detect malware. Taheri et al. [26] proposed a variety of similarity measure methods and used them for adopting different nearest neighbor algorithms that were trained using API calls, intents, and permissions extracted from multiple datasets.

Although static analysis approaches can efficiently identify malware, they may become invalid when malicious applications utilize reflection or dynamic loading to perform malicious behaviors. Dynamic analysis examines the apps during execution. It mainly focuses on the runtime behavioral analysis of apps. Hence, it can easily identify malicious behaviors that are not detected by static analysis. CrowDroid [44] used the strace tool to collect details of system calls invoked by the apps, and the 2-mean algorithm was applied to classify an app as benign or malicious. DroidScope [45] constructed OS-level and Java-level semantic views by observing changes in the environment (i.e threads and processes), system calls, and Dalvik instructions for dynamic analysis. Ng et al. [46] built a model by using a dendritic cell algorithm and regarded system calls as features. They used statistical methods to select the best features and achieve an ideal detection rate. Endroid [47] automatically extracted various kinds of dynamic behavior features, which covered system-level behavior trace and common application-level malware behaviors, such as stealing personal information, premium service subscriptions, and communication of malicious services. It adopted chi-square to remove noisy or irrelevant dynamic features and extracted critical dynamic behavior features. Yang et al. [48] proposed a malicious software detection method based on power consumption, which utilized the frequencies of energy consumption waveform to generate a Gaussian mixture model (GMM) based on Mel frequency cepstral coefficients (MFCC) to detect malicious software. Xue et al. [49] implemented a tool called Ndroid for analyzing applications with native code and can trace information leakage through multiple contexts. Vidal et al. [50] proposed a novel pattern recognition to analyze boot sequences based on analyzing dynamic behaviors of applications. MADAM [51] was a novel host-based malware detection system for android devices that identified malicious apps based on misbehavior classes. It defined different categories of malware by monitoring features belonging to four different Android levels. Wang et al. [52] developed a novel framework for mobile network traffic data to detect malware. Shabtai et al. [53] proposed Andromly, a behavior-based Android malware detection system that continuously monitors different features and patterns about the device state, such as battery level and CPU consumption. Although dynamic analysis approaches can accurately locate the malicious behavior of malware, such methods have low detection efficiency and cause huge extra overhead.

In addition, some researchers have tried to integrate dynamic analysis and static analysis to form hybrid analysis methods to improve the detection process of malware. StormDroid [54] was the first real-time analysis system developed based on streaming machine learning, and it combined static and dynamic feature extraction. Bläishing et al. [55] proposed the Android Application Sandbox (AASandbox) which detected suspicious applications by performing both static and dynamic analyses on them. ProfileDroid [56] was a hybrid analysis-based multilayer system for monitoring and profiling Android apps. It regarded permissions, intents, and java code as static features and considers user interactions, system calls, and networks as dynamic features. ANDRUBIS [57] was a fully automated large-scale and publicly available analysis system that combined static analysis along with dynamic analysis on both Dalvik VM and system level to detect Android malware. Smmarwar et al. [58] combined Random Forest and Greedy Stepwise (RF-GreedySW) to filter static and dynamic features, and then applied various machine learning algorithms to identify malware. Rasthofer et al. [59] proposed FuzzDroid, a framework that combined an extensible set of static and dynamic analyses using search-based algorithms. Wang et al. [60] first extracted dynamic API call sequences and converted them into call graphs, and then combined them with permissions to complete the identification of malware.

Recently, more detection methods have incorporated networks and deep learning (DL) architectures. DroidDetector [61] was an early detection approach that used a series of autoencoders in a deep belief network. Lee et al. [62] adopted recurrent neural networks (RNNs) and convolutional neural networks (CNNs) deep learning models for detecting malware using obfuscation techniques. DroidMalwareDetector [63] extracted permissions, API calls, and intents and converted them into word vectors, which were sent to a convolutional neural network for training to complete malware detection. Kim et al. [15] presented the first multimodal deep learning method, and existence-based and similarity-based feature extraction methods were used to process the features. Xiao et al. [64] suggested treating each system call sequence as one sentence for constructing a language that was used for training an LSTM-based deep learning classifier for identifying malware. DeepRefiner [65] also adopted LSTM with multiple hidden layers to automatically extract semantic information from the method-level bytecode of Android apps. Moreover, Pei et al. [66] utilized a word embedding representation approach which is used in the natural language processing (NLP) domain to construct a semantic feature vector that can show the relations between terms in source code. The generated semantic feature vectors were used to train a deep learning model for detecting malware and making a decision about their malware families. Chen et al. [67] extracted permissions, services, receivers and intents and fused them into a text report, and then applied BiLSTM network to mine important information in the text to complete malware detection. Pektaş et al. [68] constructed the instruction call graphs by analyzing all execution paths in Android apps, and a deep neural network model was adopted and trained to classify the apps as benign or malicious. Alzaylaee et al. [69] proposed DL-Droid, a malware detector that exploited deep learning networks. They designed a feature vector composed of 420 static and dynamic features. Although malware detection based on visualization and image processing techniques has been used widely in the desktop computer’ malware detection domain, this method has been used limitedly in Android malware detection [70]. Yen et al. [71] represented each app as an image based on its weight value, which was calculated through the importance of its instructions in the source code in the overall dataset. The constructed images were used to train a convolutional neural network, which was used to distinguish between benign apps and malicious apps. Hsien et al. [72] suggested converting the byte code of the classes.dex file of apps to RGB images with a fixed size. The generated images were used to train a convolutional neural network to classify apps as benign or malicious.

## Feature processing

After decompressing and decompiling an Android application, we can obtain byte-code, Classes.dex, and AndroidManifest.xml, which contain a large number of features such as permissions, components, and API calls. These features are diverse and large in number. Directly using these features to build a classification model will not only improve the training time and complexity of the model but also lead to many false predictions. To improve the training efficiency and detection rate of the model, the system tries to extract the security-related features and remove unnecessary features. These steps are achieved through various techniques which are discussed in further subsections.

### Feature extraction

**Fig 1:**
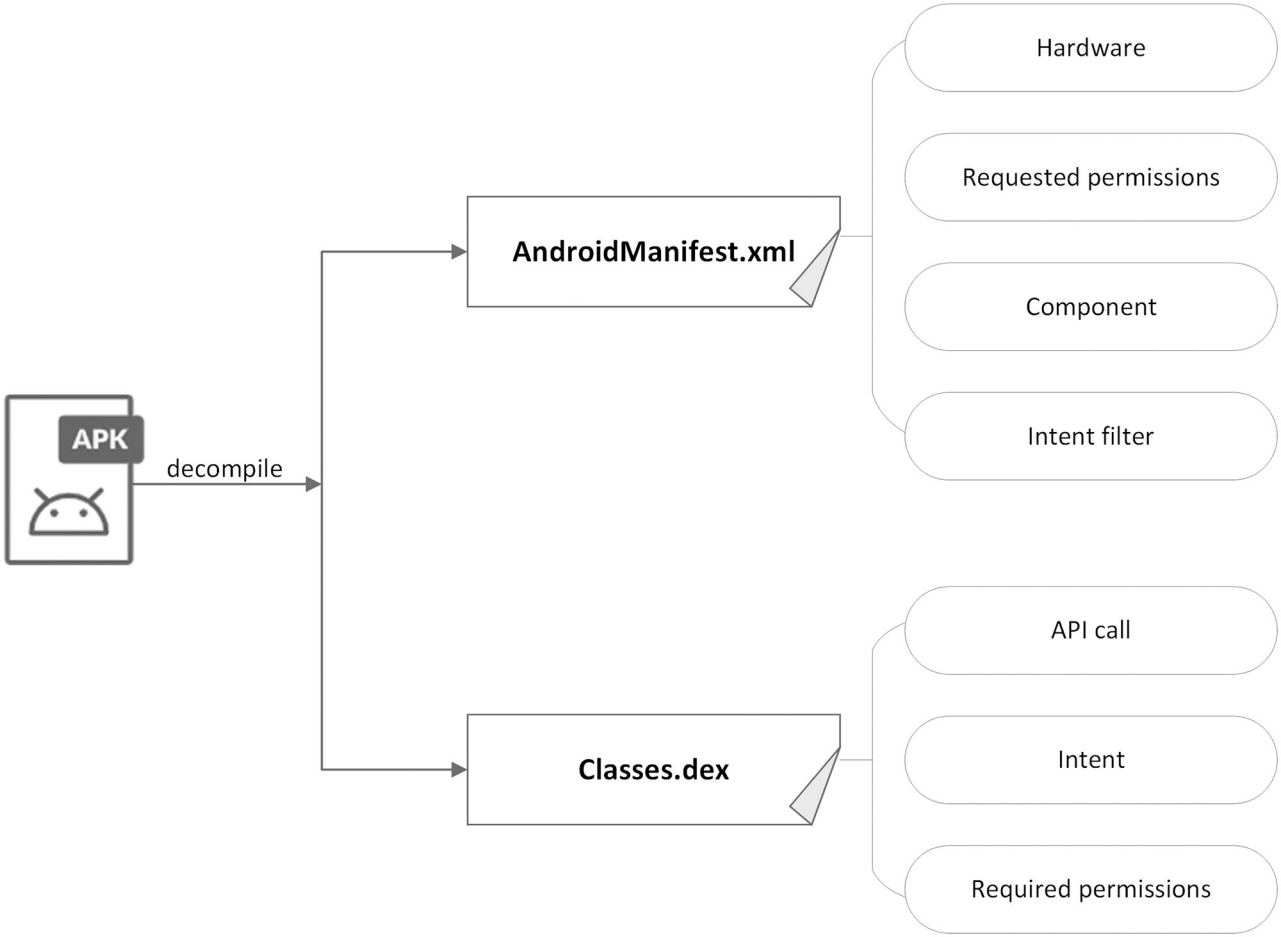
The process of feature extraction.

Android installs various applications via APK files. Androguard is an Android reverse engineering tool that can disassemble APK files. Fig 1 illustrates the process of feature extraction. By decompiling the APK file, two types of crucial files are obtained, namely, AndroidManifest.xml and Classes.dex. An AndroidManifest.xml file contains the configuration information of Android applications, such as package name, permissions, and component information, of Android applications. In the scheme, the following four categories of features are extracted.

1. Hardware: A feature that represents the hardware resources that Android applications rely on at runtime. If applications need to access the hardware resources of the Android device, such as the camera, WiFi, Bluetooth, microphone, GPS, and a variety of sensors, these features must be defined in AndroidManifest.xml accordingly.
2. Requested permissions: Android provides the permission mechanism to protect the private data of users. If applications need to access or use the resources and information of the Android device, they must declare the appropriate permissions in AndroidManifest.xml.
3. Components: Components are an essential part of Android applications and include activity, service, content provider, and broadcast receiver. They can define different interfaces and permissions that exist between applications and end-users as well as applications and the larger Android OS as a whole.
4. Intent filter: Intent filters are defined in the definition of components. It is a vital tool for inner communication between components and applications. It creates a particular filter for components and applications that can be utilized by malicious applications to wiretap on certain intents.

Then, Androguard is used to decompile the Classes.dex file, and the other three categories of features are extracted.

1. API call: Some API calls are relevant to the access of sensitive data and the underlying interaction of the Android system.
2. Required permissions: Permissions that applications use or request at runtime.
3. Intent: Intent is an abstract description of an action to be performed, and solves the inner communication between components of Android applications.

**Fig 2:**
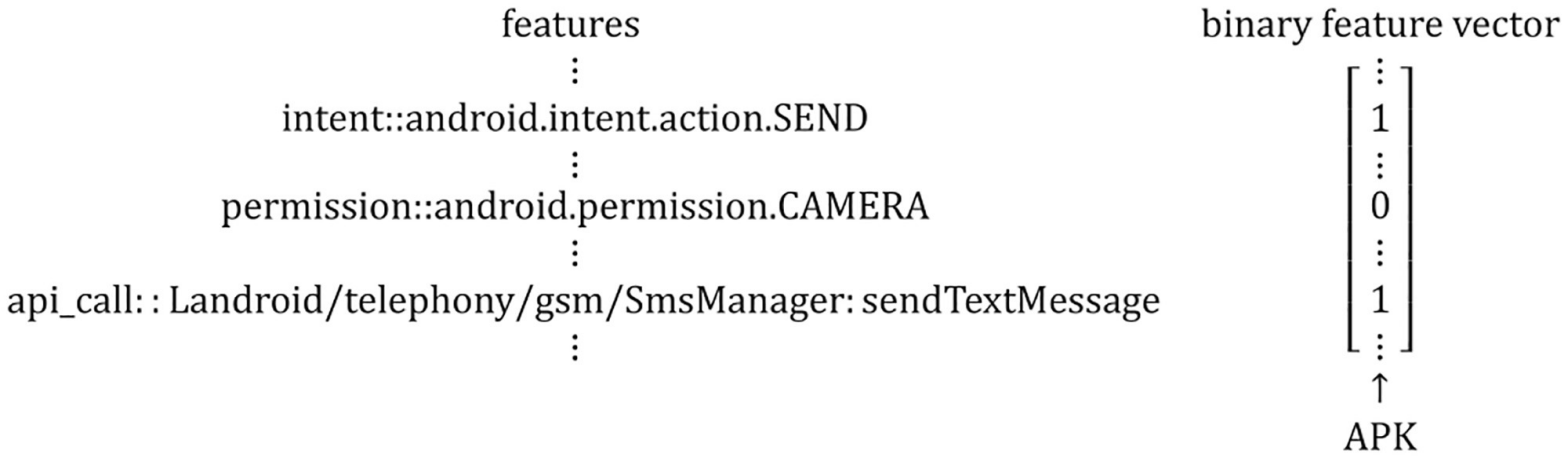
The form of feature vector.

Each feature is expressed as a string. Each application corresponds to a binary vector whose length is the same as the size of the feature set. Fig 2 demonstrates the form of the feature vector of an application. If a feature exists in an application, it is represented as 1 in the corresponding position of the feature vector, otherwise, it is marked as 0.

### Feature classification

**Fig 3:**
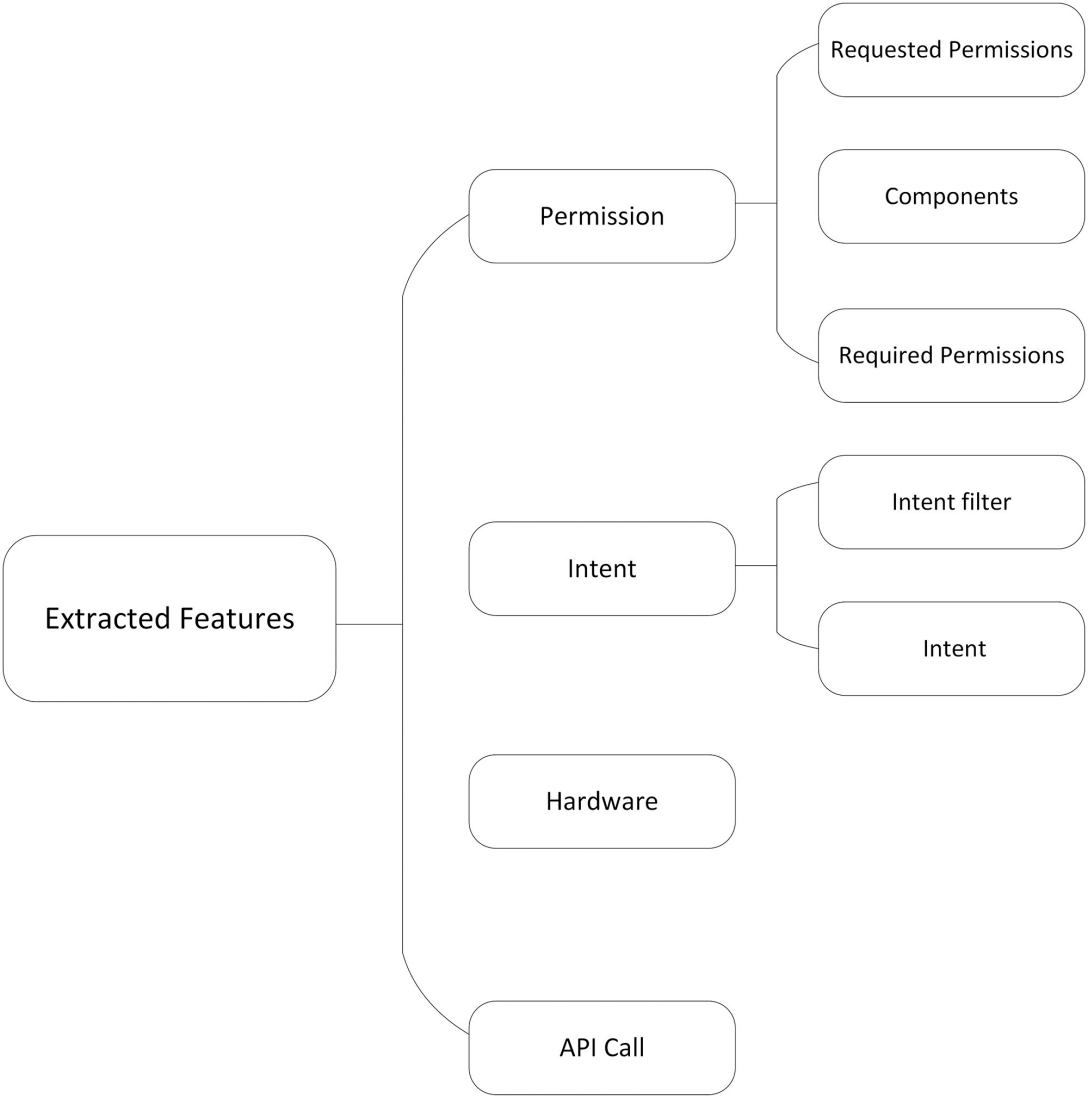
Feature classification.

After feature classification, these features are classified into four categories, as shown in Fig 3.

Google introduced a permission request mechanism in Android 6.0, and it divides all permissions into normal permissions and dangerous permissions. Applications that want to use dangerous permissions not only need to declare them in AndroidManifest.xml but also need to dynamically request them and need to be authorized by end-users. In addition, dangerous permissions are divided into groups, and if any permissions in the same group are granted, the others are automatically granted. Moreover, the component also declares some commonly used permissions, such as android.permission.BIND_DEVICE_ADMIN, to obtain administrator permissions for the device. Therefore, to obtain all permissions used by applications, the permissions declared in the AndroidManifest.xml file, the permissions in the four components, and the permissions dynamically requested and used by the application are extracted and consolidated into the permission collection used by applications. Similarly, intents declared in the AndroidManifest.xml file and dex file are combined into all intents used by applications.

### Feature selection

There are a very large number of Android applications in reality, and numerous features can be obtained from each APK file. Therefore, the number of features in the original feature set is extremely large. Directly using the entire original feature set to train the detection model will cause a very large computational overhead, and the process may suffer from “the curse of dimensionality”. In addition, many of these features may be useless or less relevant to malware identification. Consequently, it is necessary to select the most relevant features for malware identification. This is achieved through three levels of feature selection methods and is discussed next.

#### Feature selection based on the mean of weight

The features extracted from different Android applications are different. There is no need to identify and examine all features to build an accurate malware detection system. The features that are used by both benign and malware have little or no effect on distinguishing between benign and malware. Thus, they can be removed from the feature set. In addition, filter methods are not only suitable for the large-scale feature set but also have a low computational cost and can quickly remove redundant features. Therefore, a filter method called feature selection based on the mean of weight proposed in this paper is firstly used to obtain the highly distinguishable features that can be effective in distinguishing between benign and malware from the huge original feature set. The filter method needs to calculate the weight of features. Considering the different characteristics of different types of features in applications, we design two different weight calculation methods to measure the importance of features according to the characteristics of different types of features, which are called frequency similarity weight and TF-IDF difference respectively. Finally, the mean of weight of a certain type of feature is calculated as the threshold to filter the features of this type.

1. Feature importance measurement based on frequency similarity weight
  After being processed by the feature classification module, the features extracted from the application samples are finally divided into four categories, namely permission, intent, hardware, and API call. For permission, intent, and hardware, can be used by declaring in AndroidManifest.xml or by applying for authorization during the application running, that is, these three types of features are included in the application once they are declared or authorized. Therefore, for these types of features, we should not only focus on those that occur frequently in malware, but also pay attention to the low-frequency features that mainly occur in benign or malware, or even only occur in benign or malware, they are also good indicators to distinguish benign and malware. At the same time, we also should exclude features that are often used by both benign and malware, as they can interfere with malware detection. For example, almost all applications request access to the network, therefore, permission android.permission.INTERNET is frequently requested in both benign and malware, so this feature should be removed. Hence, we design a reasonable weight calculation method to measure the importance of features, called frequency similarity weight, as shown in (1)
  $$\begin{array}{c} Weight\left(f_{j}\right)=Sim\left(f_{j}\right)*|(F_{B}\left(f_{j}\right)-F_{M}\left(f_{j}\right))| \end{array}$$ (1)
  Here Weight(f_*j*_) represents the similarity weight of the j-th feature, Sim(f_*j*_) is the frequency similarity of the j-th feature in benign and malware, and F_*B*_(f_*j*_) and F_*M*_(f_*j*_) represent the frequency of j-th feature in benign and malware, respectively. Sim(f_*j*_) is calculated by (2), and its value is between [0, 1]. The closer the value of Sim(f_*j*_) is to 1, the more dissimilar the frequency of this feature is in benign and malware, which means that it is more effective for distinguishing between benign and malware. When Sim(f_*j*_)=1, the feature is only used by malware or benign, and it has an important impact on malware detection. However, when the value of Sim(f_*j*_) is 0 or close to 0, it indicates that the feature has little effect on malware identification. The frequency of each feature in malware and benign is calculated by (3) and (4).
  $$\begin{array}{c} Sim\left(f_{j}\right)=|\frac{F_{B}\left(f_{j}\right)-F_{M}\left(f_{j}\right)}{F_{B}\left(f_{j}\right)+F_{M}\left(f_{j}\right)}| \end{array}$$ (2)
  $$\begin{array}{c} F_{B}\left(f_{j}\right)=\frac{\sum_{i=0}^{N_{B}}B_{ij}}{N_{B}} \end{array}$$ (3)
  $$\begin{array}{c} F_{M}\left(F_{j}\right)=\frac{\sum_{i=0}^{N_{M}}M_{ij}}{N_{M}} \end{array}$$ (4)
  B_*ij*_ represents whether the j-th feature appears in i-th benign application, where “1” indicates that it appears and “0” indicates that it does not. M_*ij*_ represents whether the j-th feature appears in i-th malicious application. N_*B*_ and N_*M*_ represent the number of benign and malware, respectively.
  Then the mean of weight of a certain type of feature is calculated by (5), and those features whose weight is lower than the mean of weight are removed.
  $$\begin{array}{c} Mean\left(f\right)=\frac{\sum_{i=0}^{N_{f}}Weight\left(f_{i}\right)}{N_{f}} \end{array}$$ (5)
2. Feature importance measurement based on TF-IDF difference.
  For API calls, not only the frequency of API calls in samples of different categories but also their overall distribution in samples of different categories must be considered. TF-IDF can solve this problem well. The main idea of TF-IDF is that if a certain word appears in one article with high-frequency TF and rarely appears in other articles, it is considered that this word or phrase can be used to distinguish categories and is suitable for classification. The frequency of API calls in benign and malicious applications is represented by TF_*M*_(a_*j*_) and TF_*B*_(a_*j*_), as shown in (6) and (7) respectively.
  $$\begin{array}{c} TF_{M}\left(a_{j}\right)=\frac{\sum_{i=0}^{N_{AM}}a_{ij}}{N_{AM}} \end{array}$$ (6)
  $$\begin{array}{c} TF_{B}\left(a_{j}\right)=\frac{\sum_{i=0}^{N_{AB}}a_{ij}}{N_{AB}} \end{array}$$ (7)
  Where a_*ij*_ represents the number of times the j-th API call is called in i-th malware or benign. N_*AM*_ and N_*AB*_ represent the number of benign API calls and malicious API calls, respectively. The overall distribution of API calls in malware or benign is measured by IDF, as shown in (8) and (9).
  $$\begin{array}{c} IDF_{M}\left(a_{j}\right)=\frac{\sum_{i=0}^{N_{M}}M_{ij}}{N_{M}} \end{array}$$ (8)
  $$\begin{array}{c} IDF_{B}\left(a_{j}\right)=\frac{\sum_{i=0}^{N_{B}}B_{ij}}{N_{B}} \end{array}$$ (9)
  Then, the weight of the API call is calculated by (10).
  $$\begin{array}{c} Weight\left(a_{j}\right)=|TF_{dif}\left(a_{j}\right)*IDF_{dif}\left(a_{j}\right)| \end{array}$$ (10)
  Where, TF_*dif*_(a_*j*_) refers to the frequency difference of the j-th API call, and IDF_*dif*_(a_*j*_) refers to the inverse document frequency difference of j-th API call, which is calculated by (11) and (12), respectively.
  $$\begin{array}{c} TF_{dif}\left(a_{j}\right)=TF_{M}\left(a_{j}\right)-TF_{B}\left(a_{j}\right) \end{array}$$ (11)
  $$\begin{array}{c} IDF_{dif}\left(a_{j}\right)=IDF_{M}\left(a_{j}\right)-IDF_{B}\left(a_{j}\right) \end{array}$$ (12)
  Finally, the mean value of all API calls weight is calculated, and API calls whose weight is lower than the mean value are removed.

#### Correlation analysis based on pearson correlation coefficient

To further optimize the dimension of features, we analyze the remaining features and find that there are multiple groups of the same feature that always appear together, for example, android.permission.WRITE_SMS and android.permission.READ_SMS always appear together, as do android.hardware.camera and android.hardware.camera_lash. In addition, we also find that different types of features always appear in pairs. For example, android.permission.CAMERA and android.hardware.camera that always appear together, as do android.permission.CALL_PHONE and android.intent.action.CALL. Therefore, it is unnecessary to consider both features, as one of them is sufficient to characterize specific behaviors. As a result, we can retain the one with a higher frequency.

The Pearson correlation coefficient can reflect the linear correlation between two features. Thus, the correlation between two features can be measured by (13). The value of the correlation coefficient R(f_*i*_, f_*j*_) is in [-1,1], where 1 indicates a complete positive correlation, 0 indicates no linear relationship at all, and -1 indicates a complete negative correlation. Usually, a value greater than 0.8 indicates a strong correlation.

$$\begin{array}{c} R\left(f_{i},f_{j}\right)=\frac{\sum_{k=0}^{N}\left(f_{ik}-\bar{f_{i}}\right)\left(f_{jk}-\bar{f_{j}}\right)}{\sqrt{\sum_{k=0}^{N}{\left(f_{ik}-\bar{f_{i}}\right)}^{2}}\sqrt{\sum_{k=0}^{N}{\left(f_{jk}-\bar{f_{j}}\right)}^{2}}} \end{array}$$ (13)

First, the correlation analysis between features in the same category is carried out, and of two features with a correlation coefficient greater than 0.8, the one with a higher frequency is reserved. Then, the remaining features are combined to analyze the correlation between features of different types in the same way.

#### Recursive feature elimination with cross-validation

Recursive feature elimination with cross-validation (RFECV) is a wrapper method of feature selection. It removes the redundant and weak features whose deletion least affects the training error and keeps the independent and strong features to improve the generalization performance of the model. It uses an iterative procedure for feature ranking which is an instance of backward feature elimination. This technique first builds the model on the entire set of features and ranks the features according to their importance. After that, it removes the least important feature, rebuilds the model again, and recalculates the importance of features. Through RFECV, the optimal feature subset can be obtained to further reduce the dimensionality of features. However, RFECV needs to select a machine learning model to rank and select features. In this paper, we choose random forest (RF), a widely used machine learning algorithm, that not only has a fast training speed and high accuracy but can also resist noise and overfitting very well. Algorithm 1 describes the process of RFECV.

**Algorithm 1 Recursive Feature Elimination With Cross-validation.**

**Require:**

 A set of training samples with feature dimension *d*;

 A machine learning algorithm;

**Ensure:**

  The optimal feature subset *F*_*opt*_

 1: Train the ML model using all features with 5-fold cross-validation

 2: Compute the model performance

 3: Calculate the Feature importance or ranking

 4: **for** each feature subset size *F*_*i*_, *i* = 1…*d* **do**

 5:  Keep the *F*_*i*_ most important features

 6:  Train/Test model on *F*_*i*_ features

 7:  Recalculate model performance

 8:  Recalculate the importance of ranking of each feature

 9: **end for**

 10: Calculate the performance profile over the *F*_*i*_

 11: Determine the optimal number of features

 12: Select the optimal feature subset *F*_*opt*_

 13: **return** *F*_*opt*_

## Methodology

### Detection system architecture

**Fig 4:**
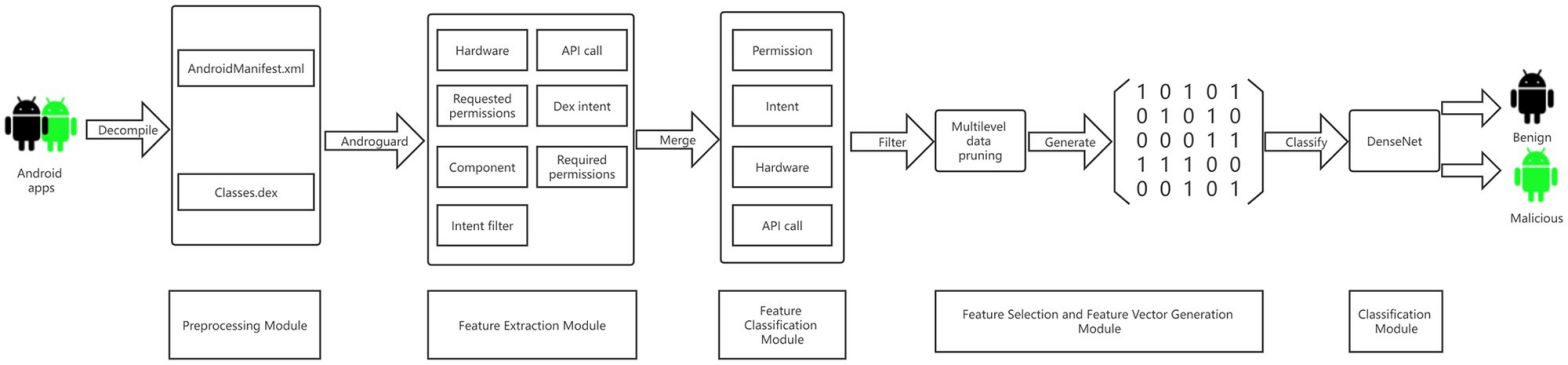
The system architecture of the proposed approach.

The overview of our designed detection approach is shown in Fig 4 and is implemented on a per-application basis. The first step involves the decompilation of the apps to obtain the intended files, which in our cases, are AndroidManifest.xml and Classes.dex. Next, Androguard is used to extract seven types of features and then combine them into four categories. After that, we develop three levels of feature selection methods to obtain the optimal feature set and generate feature vectors. Finally, the generated feature vectors as input to train DenseNet for distinguishing between benign and malicious apps.

### Main idea of DenseNet

**Fig 5:**
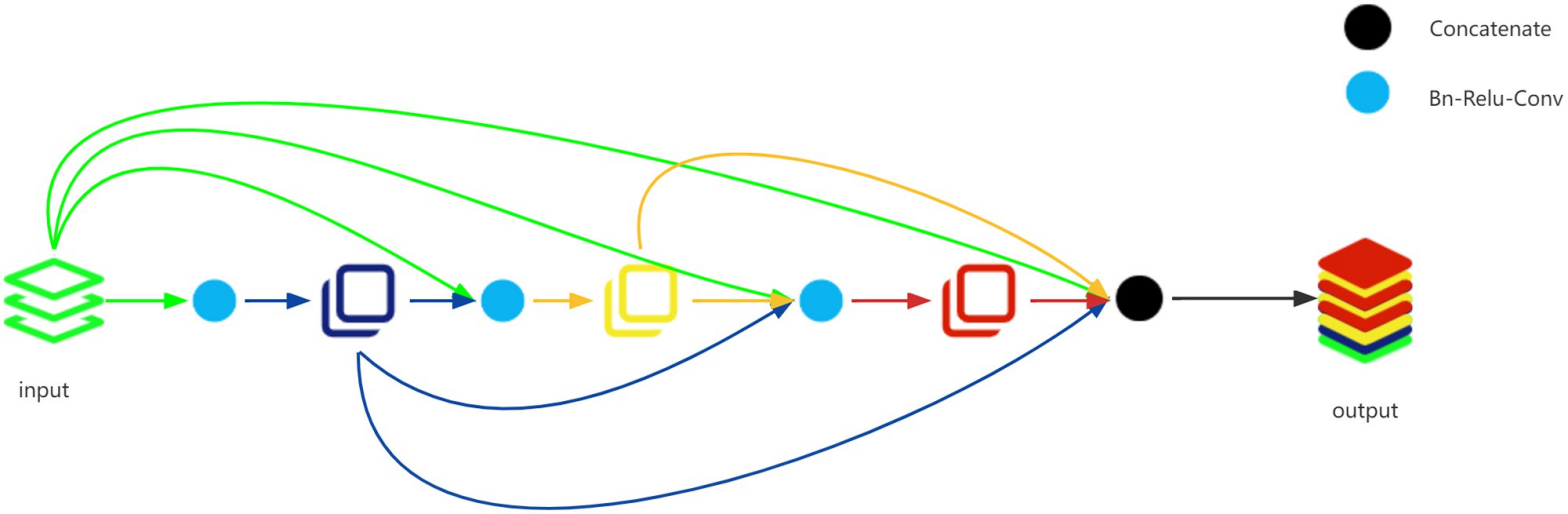
Densely concatenated convolution.

In 2017, Huang et al. [27] proposed DenseNet, which was derived from [73]. In general, the network structures are progressively hierarchical. In such a network structure, the feature maps from the (*i*-1)th layer are the input of the *i*th layer. Experiments [73] have shown that the input of the *i*th layer can be not only the output of the (*i*-1)th layer, but also the (*i*-2)th layer, or the (*i*-*n*)th layer (*n* less than the number of layers). This may lead to a more generalizable network. The basic idea of DenseNet is that each layer in the network is directly connected to the front layers. The connection strategy in DenseNet is shown in Fig 5. It is worth noting that to ensure that the feature maps can be concatenated, the sizes of feature maps need to be consistent. This means that the outputs of the convolutional layer are of the same size as the input.

The input of each layer is the output of all front layers in the densely concatenated convolution operations.

$$\begin{array}{c} X_{l}=H_{l}\left(\left[X_{0},X_{1},\cdots ,X_{l-1}\right]\right) \end{array}$$ (14)

H_*l*_ represents the *l*th layer, X_*l*_ represents the output of the *l*th layer. In DenseNet, batch normalization (BN) [74] is used to normalize the input of the layer to reduce the absolute difference between data and consider the relative difference more. The BN algorithm is described as the Algorithm 2.

**Algorithm 2 Batch Normalizing Transform.**

**Require:**

 Values of x over a batch: *B* = {*x*_1…*m*_};

 Parameters to be learned: *γ*, *β*;

**Ensure:**

  {*y*_*i*_ = *BN*_*γ*,*β*_(*x*_*i*_)}

 1: $\mu_{B}\leftarrow \frac{1}{m}\sum_{i=1}^{m}x_{i}$

 2: ${\sigma^{2}}_{B}\leftarrow \frac{1}{m}\sum_{i=1}^{m}{\left(x_{i}-\mu_{B}\right)}^{2}$

 3: ${\hat{x}}_{i}\leftarrow \frac{x_{i}-u_{B}}{\sqrt{\sigma_{B}^{2}+\varepsilon }}$

 4: $y_{i}\leftarrow \gamma {\hat{x}}_{i}+\beta \equiv {\text{BN}}_{\gamma ,\beta }(x_{i})$

More details about BN can be obtained from [74]. To increase the nonlinearity of the network, ReLU is used as the activation function and is described in (15).

$$\begin{array}{c} ReLU\left(x\right)=\{\begin{array}{cc} x & x>0\\ 0 & x\leq 0 \end{array} \end{array}$$ (15)

Compared with the other networks, the network composed of DenseNet has the following advantages.

- Fewer parameters. The input of the current layer of the network is composed of the feature maps from the front layers, which results in the continuous accumulation of feature maps. Thus, many reusable feature maps can be learned with very few convolutional kernels.
- It has a strong ability to prevent overfitting. Short paths are created from the early to late layers because of the dense connection. Each layer receives additional supervision from the loss function, which greatly reduces gradient disappearance. Therefore, the dense connection has a very good ability to resist overfitting, which makes it especially suitable for applications where training data are relatively scarce.
- Deeper layers. The network can be designed very deep by the dense connection, which only has a few parameters. For example, a network with 96 convolution layers and only 5.34 million parameters can be designed.

Experiments on ImageNet [75] demonstrate the efficiency of the dense connection [27]. Therefore, densely concatenated convolution can take full advantage of features extracted from the apps. Based on this idea, we construct a full convolution network based on DenseNet [27] for malware detection.

### Architecture of the proposed model based on DenseNet

**Fig 6:**
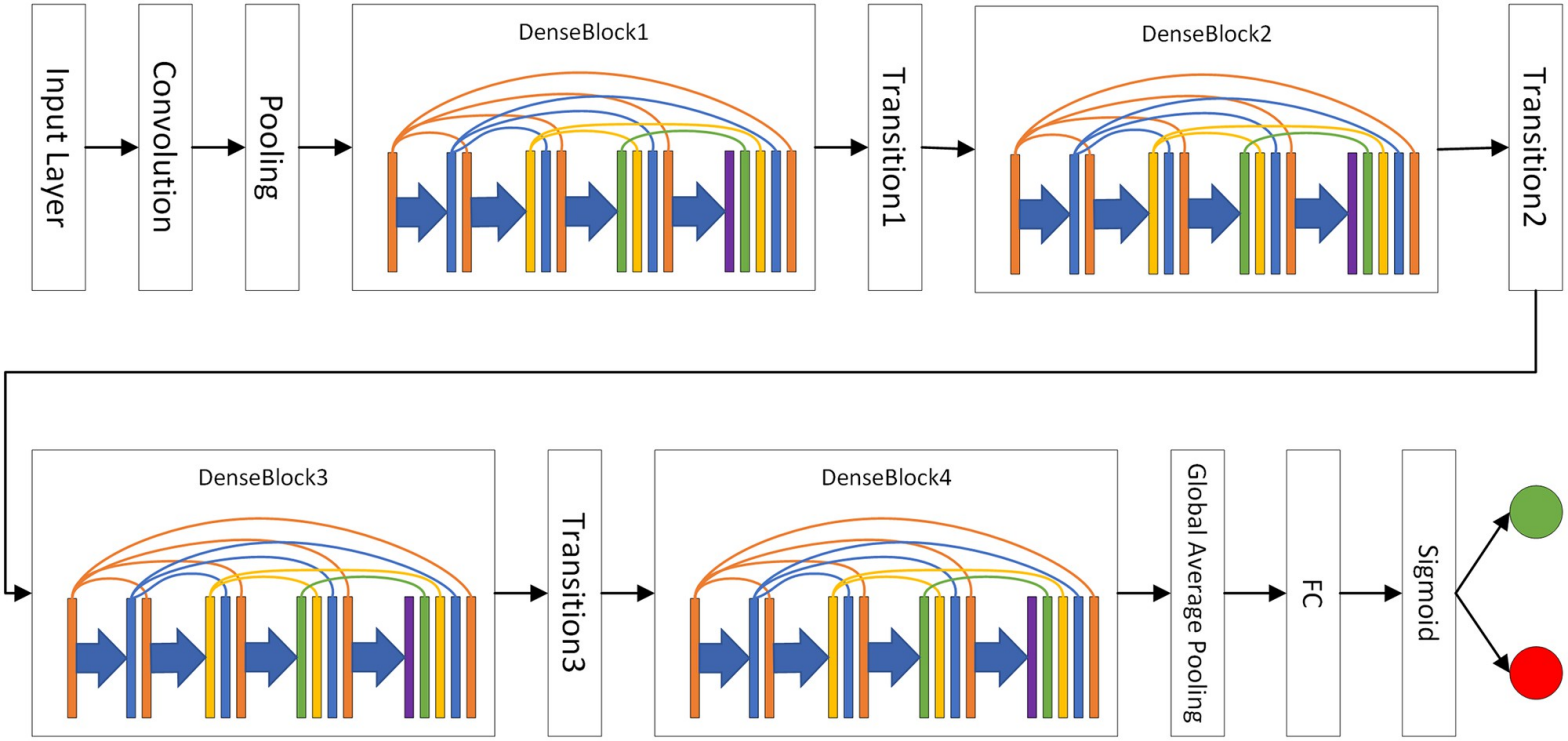
The architecture of the proposed model based on DenseNet.

An overview of our full densely connected convolutional network based on DenseNet is shown in Fig 6. The whole network is divided into an input layer, four densely connected blocks (DenseBlock), and three transition layers. After the last DenseBlock, we adopt a global average pooling operation to fix the number and size of the feature maps and then classify them by a fully connected layer with a sigmoid activation function.

As shown in Fig 6, the entire network architecture does not strictly follow the rule of dense connection; it mainly consists of densely connected blocks instead. The reason for this is that the whole network has several downsampling operations on feature maps, resulting in inconsistent of sizes between feature maps in different stages, so that feature maps with different sizes cannot be spliced in the dimension of a channel. Moreover, the concatenation operation requires feature maps to be kept in memory. With an increase in network layers, feature maps kept in memory are superimposed, which causes a very large amount of memory consumption. If every layer of the network strictly follows the dense connection theory, the network will consume many resources, which will cause the network stop working.

The parameters of our network are shown in Table 1. Our fully convolutional network mainly consists of several densely connected blocks (DenseBlock) and transition layers.

**Table 1:** The parameters of the constructed model based on DenseNet.

Layers		Input Size
Input Layer		985 × 1 × 1
3 × 3 *conv*, *s*2		985 × 1 × 1
2 × 1 *max* *pooling*, *s*2		985 × 1 × 64
Dense Block(1)	$[\begin{array}{c} 1\times 1\;conv,s1\\ 3\times 3\;conv,s1 \end{array}]\times 6\;$	985 × 1 × 64
Transition Layer(1)	$[\begin{array}{c} 1\times 1\;conv,s1\\ 2\times 1\;max\;pooling,s2 \end{array}]\;$	985 × 1 × 256
Dense Block(2)	$[\begin{array}{c} 1\times 1\;conv,s1\\ 3\times 3\;conv,s1 \end{array}]\times 12\;$	492 × 1 × 128
Transition Layer(2)	$[\begin{array}{c} 1\times 1\;conv,s1\\ 2\times 1\;max\;pooling,s2 \end{array}]\;$	492 × 1 × 512
Dense Block(3)	$[\begin{array}{c} 1\times 1\;conv,s1\\ 3\times 3\;conv,s1 \end{array}]\times 36\;$	246 × 1 × 256
Transition Layer(3)	$[\begin{array}{c} 1\times 1\;conv,s1\\ 2\times 1\;max\;pooling,s2 \end{array}]\;$	246 × 1 × 1024
Dense Block(4)	$[\begin{array}{c} 1\times 1\;conv,s1\\ 3\times 3\;conv,s1 \end{array}]\times 24\;$	123 × 1 × 512
Global Average Pooling		123 × 1 × 1024
FC,sigmoid		1 × 1 × 1024

Each DenseBlock applies one stage for extracting features and consists of multiple sets of operations, each of which includes batch normalization (BN), ReLU, Conv(1×1), and Conv(3×3). Furthermore, in all densely connected blocks, the stride of Conv(1×1) is 1 and the number of convolutional kernels is 128. Similarly, the stride of Conv(3×3) is 1, but the number of convolutional kernels is 32. To ensure that the output and input are the same size, the padding of Conv(3×3) is set to the same. It is worth mentioning that multiple sets of operations are strictly enforced in DenseBlock.

After DenseBlock, the output of each layer in the block remains in memory, resulting in a large number of feature maps. Note that if the number of these feature maps does not decrease, the network will be difficult to deepen and may stop working due to too many computations. Hence, the transition layer which consists of BN, ReLU, and Conv(1×1) is used to reduce the number of feature maps to half. In addition, it can not only reduce the number of feature maps but also increase the depth and improve the fitting ability of the network.

To further reduce the computational consumption, a global average pooling layer is used after four densely connected blocks and three transition layers to further reduce the number of feature maps without increasing the network parameters.

Finally, a fully connected layer with a sigmoid activation function is used for classification. Furthermore, the sigmoid activation function is widely used in binary classification, as shown in (16). The value of S(x) represents the probability that the sample is a positive class. In addition, the Adam optimizer is used, and a dropout rate of 0.2.

$$\begin{array}{c} S\left(x\right)=\frac{1}{1+e^{-x}} \end{array}$$ (16)

## Evaluation

In this section, we evaluate our work from the following four aspects.

- The performance of three levels of feature selection methods.
- The performance improvement obtained by our method compared to common machine learning models.
- The performance of our detection method compared to that of two state-of-the-art neural networks.
- The performance of our detection method compared to other similar detection approaches.

Before performing these evaluations, this section first introduces the dataset and metrics and then describes the details of feature processing.

### Data collection

The dataset consists of 15000 benign apps and 15000 malicious apps. Benign apps were crawled from Google Play and include different categories, such as social, news, entertainment, finance, education, game, sport, music, shopping, banking, and weather apps. Each app is submitted to virustotal [76], an online scan engine to verify that legitimate files are not injected with the malicious payload. Malicious apps were collected from VirusShare. The details of the dataset are shown in Table 2.

**Table 2:** The details of the dataset.

Datasets	Source	Number of apps	Number of features
Malware	VirusShare	15000	20174
Benign	Google Play	15000	25229

### Evaluation metrics

Malware detection is a classification problem. For classification problems, six metrics are usually used for evaluation, namely, accuracy, precision, recall, F_1_-score, the true positive rate (TPR), and the false positive rate (FPR). Malicious samples are denoted as the positive (P) class and benign samples are denoted as the negative (N) class. Afterward, four definitions are obtained.

#### True positive (TP)

The number of positive samples that are correctly predicted to be positive.

#### False positive (FP)

The number of negative samples that are wrongly predicted as positive.

#### True negative (TN)

The number of negative samples that are correctly predicted as negative.

#### False negative (FN)

The number of positive samples that are wrongly predicted as negative.

Based on these definitions, the six metrics are calculated as follows.

$$\begin{array}{c} Accuracy=\frac{TP+TN}{TP+FP+TN+FN} \end{array}$$ (17)

$$\begin{array}{c} Precision=\frac{TP}{TP+FP} \end{array}$$ (18)

$$\begin{array}{c} Recall=\frac{TP}{TP+FN} \end{array}$$ (19)

$$\begin{array}{c} F_{1}-Score=\frac{2*Precision*Recall}{Precision+Recall} \end{array}$$ (20)

$$\begin{array}{c} TPR=\frac{TP}{TP+FN} \end{array}$$ (21)

$$\begin{array}{c} FPR=\frac{FP}{FP+TN} \end{array}$$ (22)

The proposed method is implemented in the Python programming language (version 3.6). Moreover, multiple well-known Python libraries, such as sklearn, Keras, and TensorFlow, are used to develop the presented models. All the experiments run on a personal computer with an Intel (R) Core(TM) i7–9750H @2.86 Hz CPU with 16 GB of memory, an NVIDIA Geforce GTX 1660Ti, and a Windows 10 64-bit operating system.

### Details of feature processing

Table 2 indicates that 20,174 features are extracted from malicious apps and 25,229 features are extracted from benign apps. However, some of the features are found in both benign and malicious applications, so a total of 32,801 features are obtained after deduplication. The details of each category of features are shown in Table 3. Among them, the number of API calls is the largest, which is 18612; the number of hardware is the least, which is 150; and the number of permissions and intents are 2374 and 11665, respectively.

**Table 3:** The details of the extracted features.

Datasets	Number of permissions	Number of intents	Number of API calls	Number of hardware
Malware	1113	5714	13292	55
Benign	1668	6944	16477	140
Total	2374	11665	18612	150

It can be seen that the number of features in the original feature set is enormous. Hence, a fast filter method called feature selection based on the mean of weight, abbreviated as FSMW is applied at the first level to obtain highly distinguishable features from a large number of features. In this method, we design two weight calculation methods, namely frequency similarity weight and TF-IDF difference. Then, the mean of weight of a certain type of feature is calculated as the threshold, and the features whose weight is lower than the threshold are removed from the feature set. The feature information after FSMW processing is displayed in Table 4. After being processed by FSMW, the number of permissions and intents decreased by 92%, the number of Hardware decreased by 84%, and the number of API calls decreased by 74%. Now, the number of features is 5990, which is 81% less than the number of the original feature set.

**Table 4:** The details of the features processed by FSMW.

Feature	Approach	Threshold	Number
Permission	Frequency similarity weight	0.007	179
Intent	Frequency similarity weight	0.0014	963
Hardware	Frequency similarity weight	0.006	23
API call	TF-IDF difference	0.00003	4825

The top ten permissions of the frequency similarity weight value are shown in Table 5. It can be seen that the top ten permissions include not only the high-risk permissions defined by Google that are frequently used by malware; for example, the permission SEND_SMS is used to send a message, the permission RECEIVE_SMS is used to receive a message, and the permission READ_SMS is used to read a message. Some permissions are used almost exclusively in malware; for example, the permission WRITE_SETTINGS is used to write system settings, the permission INSTALL_SHORTCUT is used to remove application shortcuts, the permission MOUNT_UNMOUNT_FILESYSTEMS is used to create or delete files on an sd card, the permission SYSTEM_ALERT_WINDOW is used to display the system window, the permission RECEIVE_BOOT_COMPLETED is used to self-start at boot, and the permission BIND_DEVICE_ADMIN is used to obtain the super administrator of a device. In addition, the common permission CHANGE_WIFI_STATE, which mainly occurs in benign apps, is also included. Therefore, the proposed weight method not only focuses on the features that are frequently used in benign or malware but also focuses on the distinction between benign and malware. Moreover, the top ten intents and hardware of the frequency similarity weight value are shown in Tables 6 and 7, respectively. The top ten API calls of TF-IDF difference are shown in Table 8.

**Table 5:** The top ten permissions of the frequency similarity weight value.

android.permission.SEND_SMS
android.permission.RECEIVE_SMS
android.permission.READ_SMS
android.permission.WRITE_SETTINGS
com.android.launcher.permission.INSTALL_SHORTCUT
android.permission.MOUNT_UNMOUNT_FILESYSTEMS
android.permission.SYSTEM_ALERT_WINDOW
android.permission.RECEIVE_BOOT_COMPLETED
android.permission.CHANGE_WIFI_STATE
android.permission.BIND_DEVICE_ADMIN

**Table 6:** The top ten intents of the frequency similarity weight value.

android.intent.extra.REFERRER_NAME
android.intent.category.LEANBACK_LAUNCHER
com.android.vending.INSTALL_REFERRER
android.app.action.DEVICE_ADMIN_ENABLED
android.intent.action.INSERT
android.intent.action.SEARCH
android.intent.action.BOOT_COMPLETED
android.content.intent.ACTION_USER_UNLOCKED
android.intent.action.BATTERY_CHANGED
android.intent.action.DIAL

**Table 7:** The top ten hardware of the frequency similarity weight value.

android.hardware.camera.front
android.hardware.telephony
android.hardware.microphone
android.hardware.screen.portrait
android.hardware.touchscreen.multitouch
android.hardware.nfc.hce
android.hardware.bluetooth_le
android.hardware.leanback
android.hardware.vulkan
android.hardware.autofocus

**Table 8:** The top ten API calls of TF-IDF difference.

getSimSerialNumber
getSubscriberId
getLine1Number
getNetWorkOperatorName
getNetworkType
getExternalStorageDirectory
getLastKnownLocation
getSimOperator
getLongitude
getExternalFilesDir

After that, a correlation analysis based on the Pearson correlation coefficient, abbreviated as CAPCC, is used to further reduce the number of features. First, a correlation analysis between features in the same category is carried out, and then a correlation analysis between features in different categories is carried out. When the value of the Pearson correlation coefficient is greater than 0.8, it indicates that there is a strong correlation between two features, so we set the threshold value as 0.8. Some of the selected feature pairs with strong relationships are shown in Table 9. The less frequent features in feature pairs with strong relationships are subsequently removed from the feature set. The details of features processed by CAPCC are shown in Table 10. After the correlation analysis between the same categories of features, the number of features is reduced to 2424, of which the number of permissions is 111, the number of intents is 267, the number of hardware is 19, and the number of API calls is 2027. Furthermore, through the correlation analysis between features of different categories, the number of features becomes 2382, a further decrease of 53%.

**Table 9:** Some of the selected feature pairs with strong relationships.

android.permission.SEND_SMS	android.permission.WRITE_SMS
android.hardware.camera	android.hardware.camera.flash
android.intent.extra.BCC	android.intent.extra.CC
getLongitude	getLatitude
android.permission.BLUETOOTH	android.hardware.bluetooth
android.permission.CALL_PHONE	android.intent.action.DIAL
android.permission.ACCESS_FINE_LOCATION	getCellLocation

**Table 10:** The details of the features processed by CAPCC.

Feature	Type	Threshold	Number
Permission	same	0.8	111
Intent	same	0.8	267
Hardware	same	0.8	19
API call	same	0.8	2027
Total	different	0.8	2382

Finally, RFECV, a wrapper approach, is adopted to obtain the optimal feature subset of the preserved features. Moreover, the details of the features processed by RFECV are shown in Table 11. We choose random forest (RF), a widely used algorithm in machine learning. In addition, we set the cross-validation parameter to 10, that is, 10-fold cross-validation. Through RFECV, the final feature set number is 985, which is approximately 96% less than the number of original features.

**Table 11:** The details of the features processed by RFECV.

Feature	ML	K-Fold	Number
Permission	RF	10	65
Intent	RF	10	87
Hardware	RF	10	18
API call	RF	10	815

Table 12 shows the quantitative information of each feature after three levels of feature methods. The number of features processed by our designed three levels of feature selection methods continues to decrease. Next, we will verify the effectiveness of our proposed three levels of feature selection methods through experiments.

**Table 12:** The details of the features processed by three levels of feature selection methods.

Approach	Number of permissions	Number of intents	Number of API calls	Number of hardware
FSMW	179	963	4825	23
CAPCC	111	267	2027	19
RFECV	65	87	815	18

### Evaluation of three levels of feature selection methods

In this experiment, to evaluate the performance of the proposed three levels of feature selection methods, we use logistic regression (LR), a machine learning algorithm with small computations and high speed, to evaluate the detection performance of the features at each stage. The dataset is split into 70% for training and 30% for testing. Table 13 shows the influence of feature sets on execution time, precision, and accuracy after being processed by three levels of feature selection methods. It can be seen that the training time of the feature set processed by the three pruning methods is 100 times less than that of the original feature set, while the accuracy is only 0.45% lower. Therefore, our proposed three levels of feature selection methods are effective.

**Table 13:** The influence of three levels of feature selection methods on the effect of feature set.

Approach	Number of features	Training time	Precision	Accuracy	TPR
Original	32801	119.18s	99.90%	99.93%	99.96%
FSMW	5990	17.98s	99.90%	99.91%	99.96%
FSMW+CAPCC	2382	4.25s	99.87%	99.88%	99.90%
FSMW+CAPCC+RFECV	985	1.63s	99.60%	99.48%	99.48%

### Comparison with machine learning algorithms

In this experiment, to further verify the validity of the selected features, we use a variety of machine learning algorithms for training and testing. In addition, we also compare the proposed method with these machine learning algorithms to verify the effectiveness of our method. The performance of our method and these machine learning algorithms are shown in Table 14. Five common machine learning algorithms are used in this experiment: logistic regression (LR), support vector machine (SVM), random forest (RF), extremely randomized tree (ET), and decision tree (DT). According to the experimental results, The optimal feature subset obtained by our proposed three levels of feature selection methods achieves good results on all five machine learning algorithms, and even the worst algorithm, DT, has an accuracy of 99.13%, which further proves the effectiveness of our proposed three levels of feature selection methods. In addition, Our detection method outperforms these five machine learning methods on all evaluation metrics. Our detection method improves the accuracy by 0.7% compared to the worst machine learning algorithm DT and 0.23% compared to the best machine learning algorithm SVM. As a consequence, our detection method based on deep learning is superior to these traditional machine learning methods in performance. In addition, our method has the lowest FPR of 0.15%.

**Table 14:** The comparison with machine learning algorithms.

Model	Precision	Recall	F1-score	Accuracy	TPR	FPR
LR^a^	99.60%	99.48%	99.54%	99.48%	99.48%	0.51%
SVM^b^	99.63%	99.66%	99.65%	99.60%	99.66%	0.47%
RF^c^	99.51%	99.66%	99.59%	99.53%	99.66%	0.62%
ET	99.54%	99.75%	99.65%	99.60%	99.75%	0.58%
DT	99.15%	99.30%	99.22%	99.13%	99.30%	1.09%
Ours	99.88%	99.82%	99.85%	99.83%	99.82%	0.15%

^a^The value of the parameter max_iter of LR is 3000

^b^The parameter kernel of SVM is linear

^c^The values of parameters n_estimators, min_samples_leaf, and n_jobs in RF are 750, 2, -1 respectively

**Fig 7:**
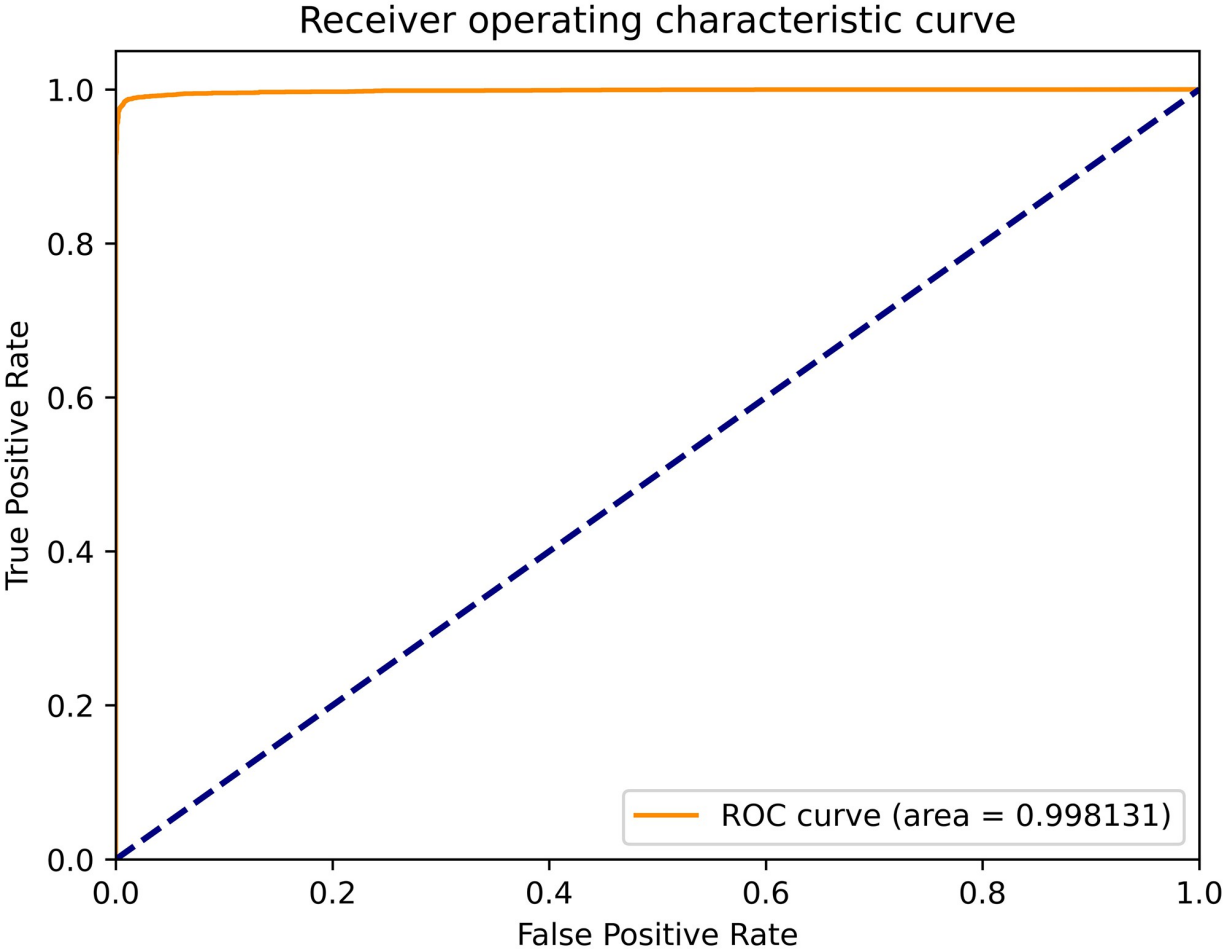
The ROC curve of our method.

**Fig 8:**
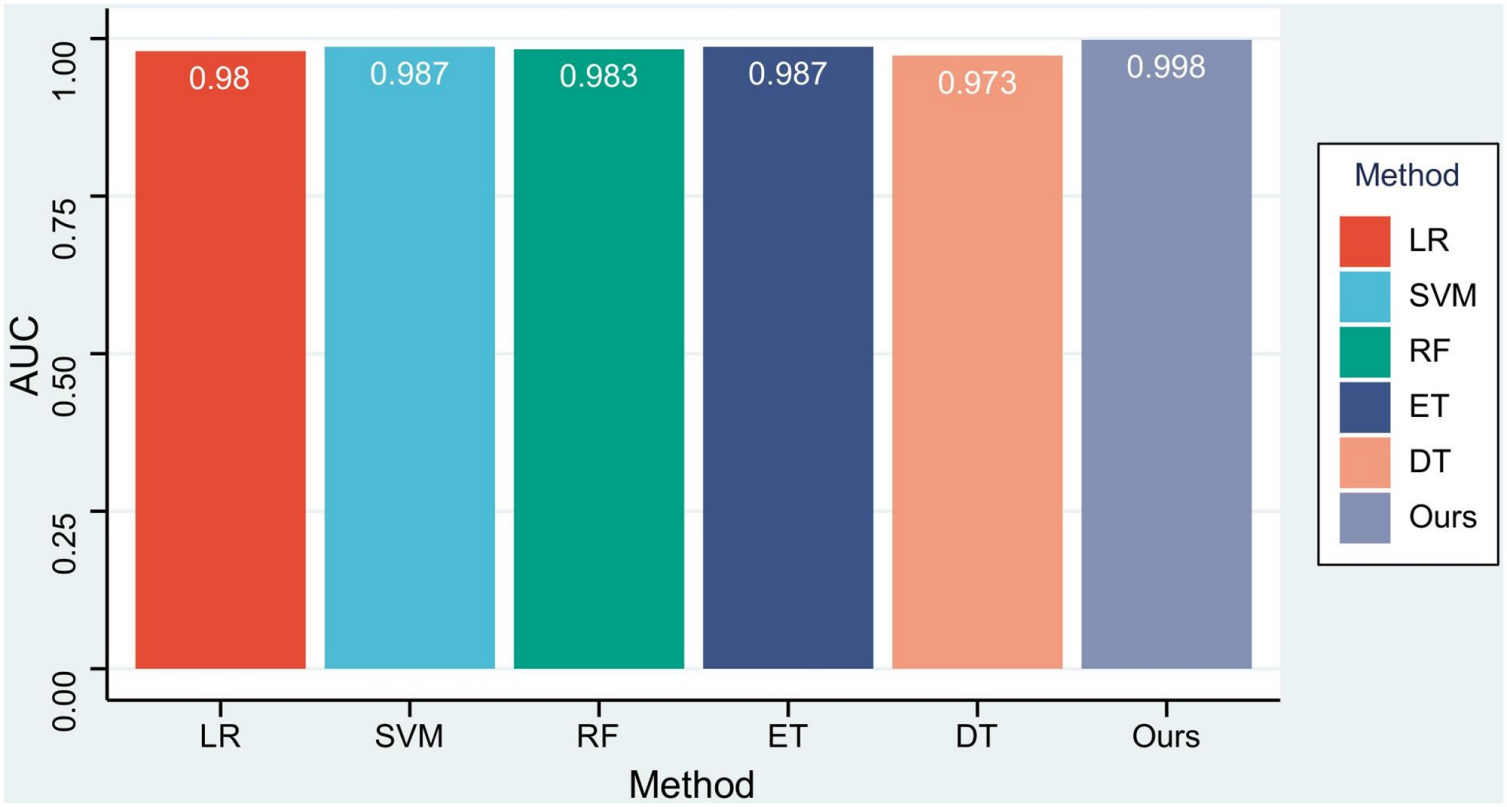
The AUCs of our method and machine learning algorithms.

The receiver operating characteristic (ROC) curve and area under the curve (AUC) are often used to evaluate the merits of a binary classifier. The ROC curve of our method is shown in Fig 7. As shown in Fig 7, the AUC of our method is 0.998, and a comparison with the AUCs of the selected machine learning algorithms is shown in Fig 8. It can be seen that the AUC of our method is the highest, which means that our method is the best.

### Comparison with well-known deep learning models

In this section, we select two well-known deep learning models ResNet and VGG16 for training and testing and compare their performance with that of the proposed deep learning model based on DenseNet.

ResNet [51], short for residual network, is a typical neural network that was proposed to allow training extremely deep neural networks while maintaining the computational cost at an acceptable level. ResNet depends on Skyping over unimportant layers or the layers that initially do nothing, which can enable faster training.

The second model used is VGG16 [52], a deep CNN network proposed by the Visual Geometry Group (VGG) in 2015. They improved performance by increasing the number of network layers and used architecture with small convolution filters of size 3 × 3 to reduce the number of parameters.

We used 800 epochs, the Adam optimizer, the sigmoid activation function, and a dropout rate of 0.2 in each neural network. In all comparison experiments, 60% of the dataset was used for training, 20% of the dataset was used for validation, and 20% of the dataset was used for testing. The computational overhead and the number of parameters of each neural network are shown in Table 15. Compared with VGG16 and ResNet, the proposed deep learning model based on DenseNet uses the fewest parameters and takes the least training time.

**Table 15:** The computational overhead and the number of parameters of each model.

Model	Training time of each epoch	Number of parameters
Our	15s	6864961
ResNet	35s	45740801
VGG16	22s	22852033

**Fig 9:**
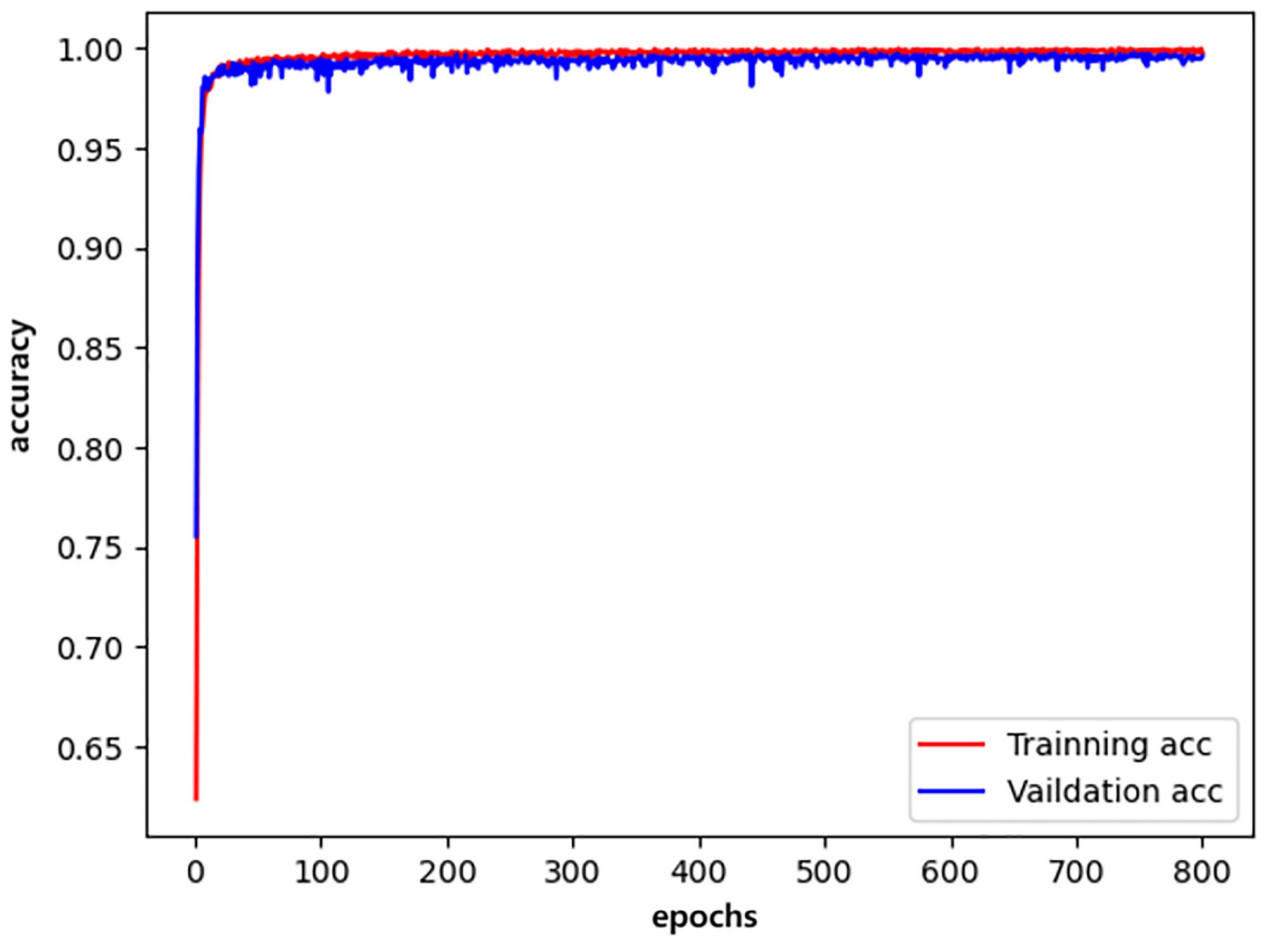
densenet_accuracy.

**Fig 10:**
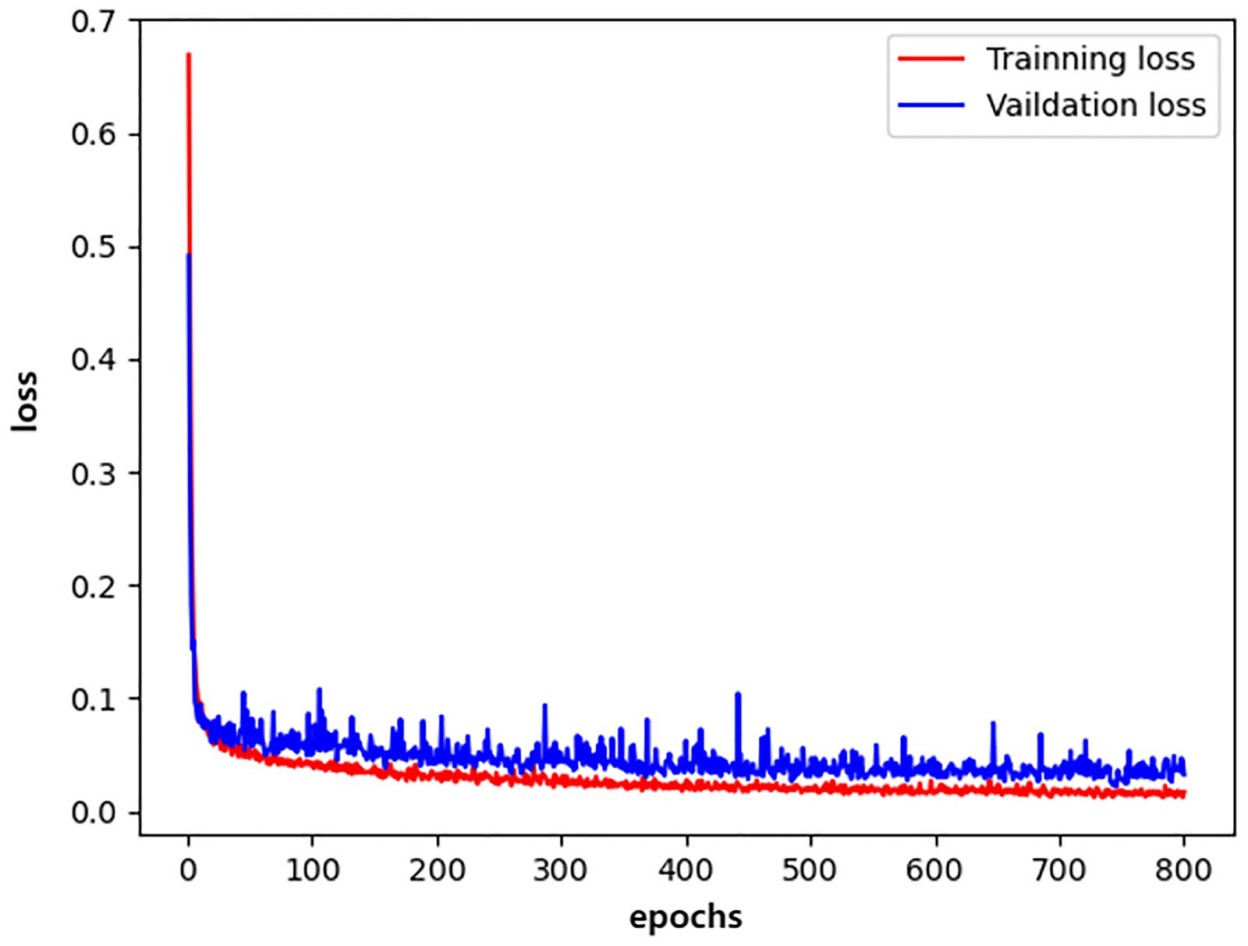
densenet_loss.

**Fig 11:**
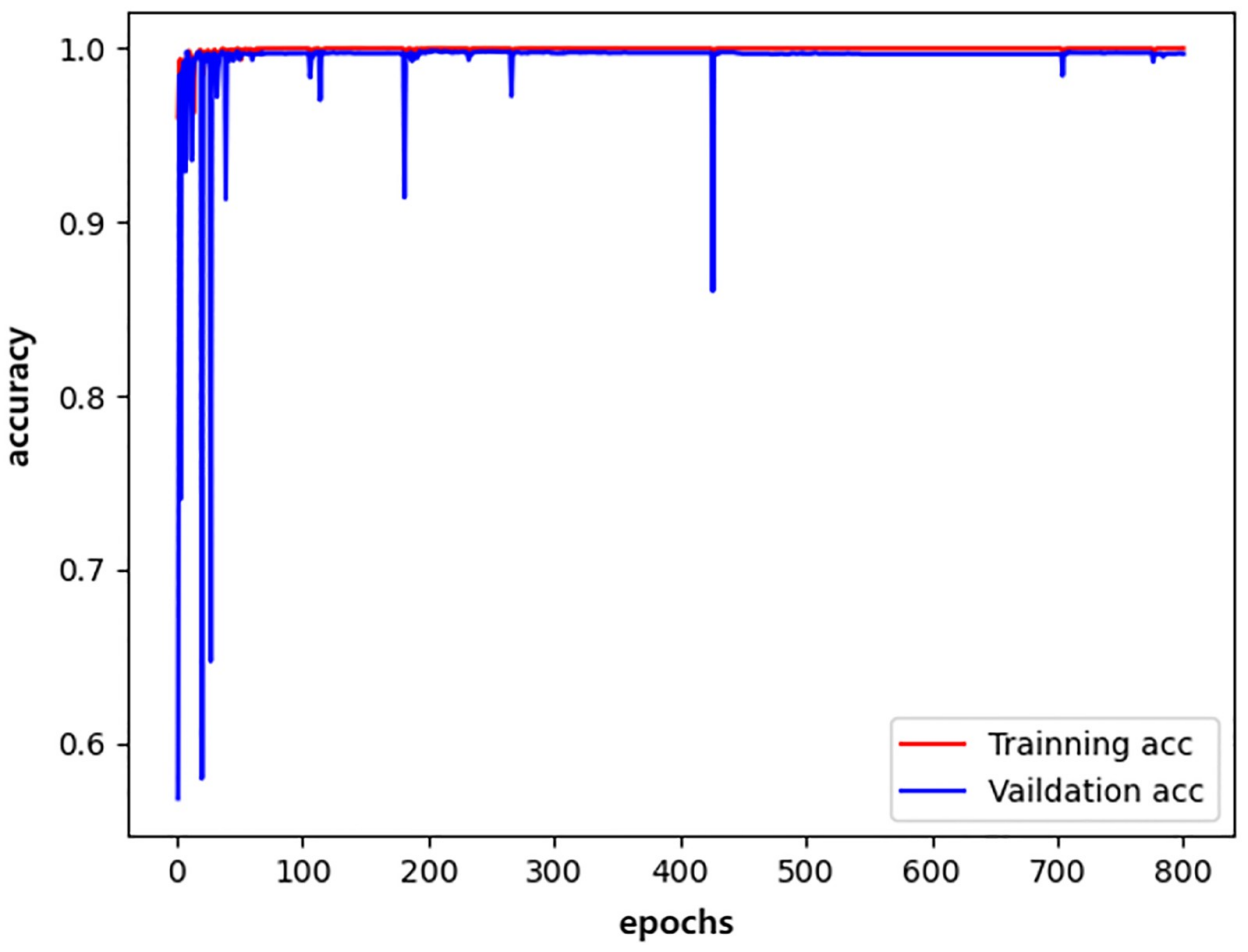
resnet_accuracy.

**Fig 12:**
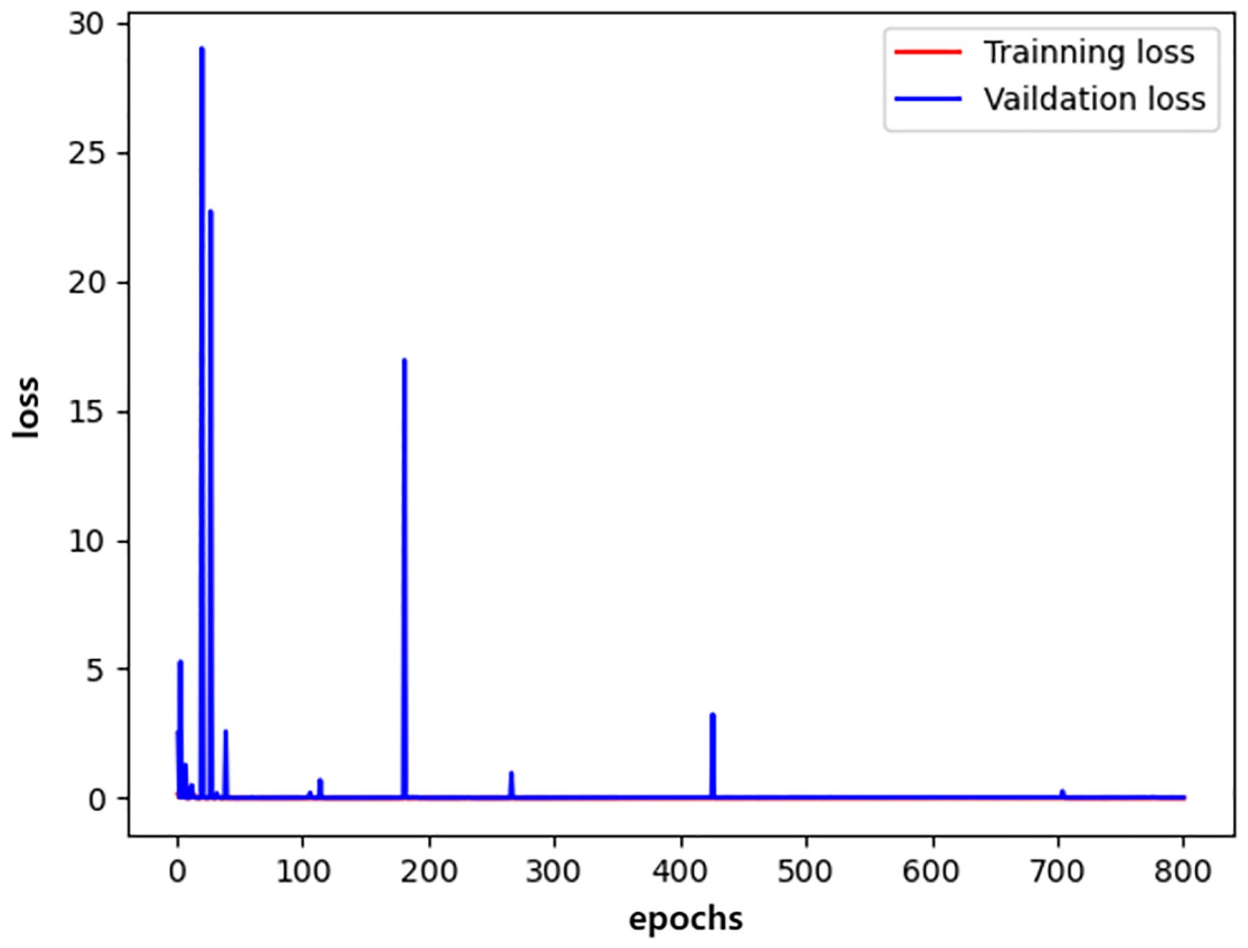
resnet_loss.

**Fig 13:**
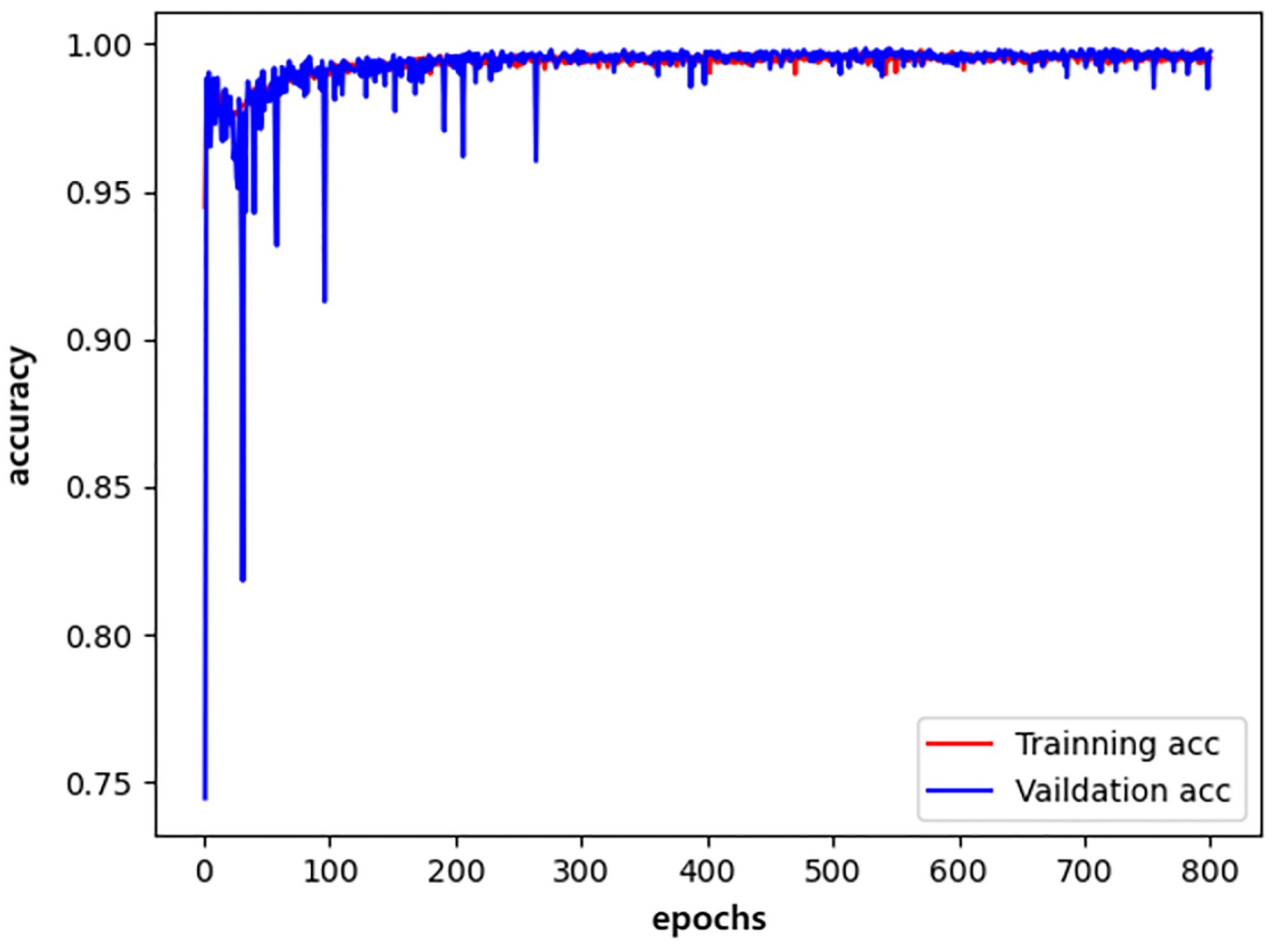
vgg16_accuracy.

**Fig 14:**
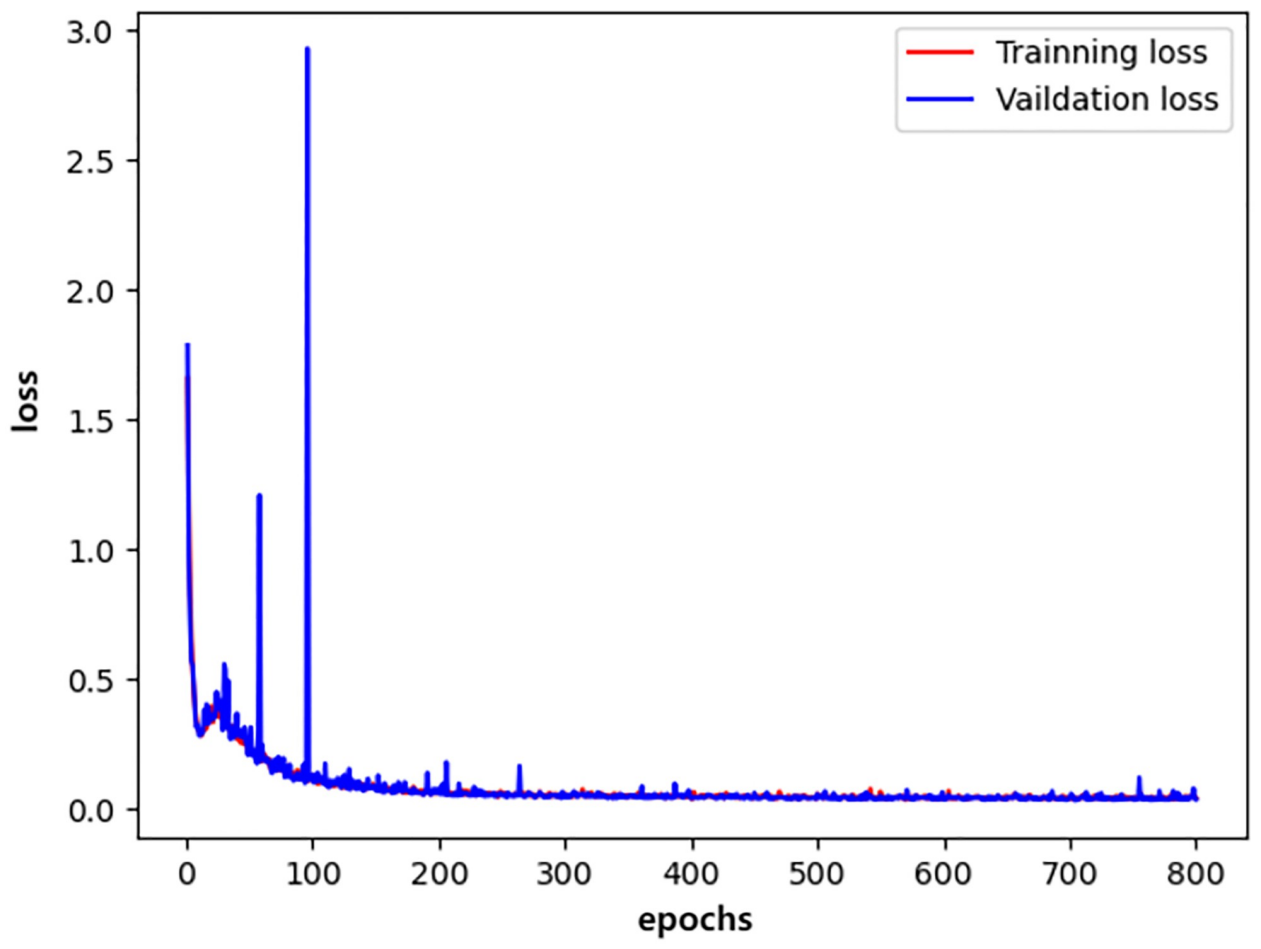
vgg16_loss.

We plotted the changes in the training accuracy, validation accuracy, training loss, and validation loss during the 800 epochs of the three models in all experiments as illustrated in Figs 9–14.

Generally, the validation dataset is used to adjust the model parameters and verify the generalization ability of the model. Therefore, we save the model with the smallest verification loss for each network and use it for testing on the test set. The results obtained by the networks with the smallest validation loss are shown in Table 16. It can be noticed that our detection method achieves good detection performance while reducing model parameters and computing overhead. Furthermore, an accuracy of 99.76% is achieved on the validation dataset and an accuracy of 99.72% on the test dataset.

**Table 16:** The performance of each neural network.

Model	Training accuracy	Training loss	Validation accuracy	Validation loss	Testing accuracy	Tesing loss
Ours	99.96%	0.017	99.76%	0.02255	99.72%	0.03237
ResNet	99.99%	0.003	99.80%	0.00772	99.75%	0.01243
VGG16	99.92%	0.025	99.82%	0.03428	99.69%	0.03942

### Comparision with existing methods in detection performance

In this section, we compare our method with other previously proposed and similar detection approaches on the Drebin dataset. The detection performance of our proposed method and the comparison methods on the Drebin dataset are shown in Table 17. As shown in Table 17, for the three evaluation indicators of accuracy, TPR and FPR, our method obtains better results than the comparison methods. In addition, our method extracts various types of features to reflect all aspects of applications as comprehensively as possible. Our designed multiple feature selection methods solve the problem of excessively large feature sets caused by various types of features and select features with high discrimination abilities.

**Table 17:** The performance of each neural network.

Methods	Model	Feature diversity^a^	Feature dimension^b^	Accuracy	TPR	FPR
Ours	DenseNet	High	Medium	99.86%	99.82%	0.15%
Arp [40]	SVM	High	High	93.90%	NA	1.00%
Xu [77]	SVM	Low	Low	93.10%	97.40%	0.67%
Bai [78]	CatBoost	High	Medium	97.40%	96.77%	1.97%
Grosse [79]	CNN	High	Medium	98.35%	NA	1.29%
Salah [80]	SVM	High	Medium	99.00%	NA	NA
Yusof [81]	RF	Low	Low	NA	99.40%	16.10%
Zhang [23]	CNN	High	High	97.40%	98.30%	3.20%

^a^In the definition of feature diversity, Low refers to when the number of feature categories is 1 or 2, Medium refers to when the number of feature categories is 3 or 4, and High refers to when the number of feature categories is more than 4.

^b^In the definition of feature dimension, when the number of features is less than 500, it is defined as Low, when the number of features is between 500 and 5000, it is defined as Medium, and when the number of features is greater than 5000, it is defined as High.

## Conclusion

With the increasing number of applications that can be classified as malware and the advancement of malware types and camouflage techniques, the effective detection of malware in a relatively short period of time is of considerable importance to the third-party application market and users. How to improve the detection accuracy and reduce the detection time is still an urgent problem to be solved.

In this paper, we present a novel Android malware detection framework, that utilizes several static features to reflect the properties of applications in various aspects. By analyzing the manifest file and dex file, seven types of static features are extracted and these features enrich the extracted information to express the behaviors of applications. In addition, to screen out features that can be used to effectively distinguish benign applications from malicious applications and reduce the feature dimension to reduce the computational overhead, we propose three levels of feature selection methods to obtain the highly distinguishable features that can be effective in distinguishing between benign and malware. Finally, we construct a fully convolutional network based on DenseNet for training and prediction using the selected features. In the evaluation, we carried out four experiments. First, we demonstrated the effectiveness of the proposed three levels of feature selection methods. The experimental results show that the number of features in the original feature set decreases from 32801 to 985, which greatly reduces the training time of the model with only 0.45% accuracy loss. Then, we performed experiments to prove the effectiveness of our proposed deep learning model by comparing it with traditional machine learning models. The five machine learning algorithms with the obtained optimal feature subset achieve good results, and DT, the worst algorithm, still achieves an accuracy of 99.13%. In addition, the proposed detection method is superior to the five machine learning methods in all evaluation indicators. Afterward, we conduct experiments with two well-known deep learning models, ResNet and VGG16, to compare the performance of the proposed method. The proposed detection method achieves a good detection effect with 99.72% accuracy while greatly reducing the training cost. Finally, we compare our method with other detection approaches on the Drebin dataset. As a result, the proposed method is superior to the comparison methods in all evaluation indicators. The false positive rate of our method is only 0.15%, 15.95% lower than that of the comparison method with the highest false positive rate. Hence, our framework is effective enough to be used for Android malware detection.

Different categories of features reflect the different behaviors of applications, so a rich variety of features can describe the behaviors of applications more comprehensively. Through an in-depth exploration of features related to the behaviors of applications, we finally extract seven types of static features to express the behaviors of applications. In future work, we will continue to conduct in-depth research on features related to the behavior of applications, and constantly add new categories of key static features to describe the behaviors of applications more comprehensively, and to achieve a more accurate detection effect. In addition, since our detection method uses static analysis for speed and accuracy, it cannot solve the problems of reflection and dynamic loading. Therefore, in future work, we will consider introducing key dynamic features into the feature set to solve the problems that cannot be solved by static analysis such as reflection and dynamic loading, while ensuring the detection speed as much as possible.

## Supporting information

S1 Data (CSV)

S2 Data (CSV)

S3 Data (CSV)
